# Chalcogenide optomemristors for multi-factor neuromorphic computation

**DOI:** 10.1038/s41467-022-29870-9

**Published:** 2022-04-26

**Authors:** Syed Ghazi Sarwat, Timoleon Moraitis, C. David Wright, Harish Bhaskaran

**Affiliations:** 1 IBM Research- Europe, Säumerstrasse, 8803 Rüschlikon, Switzerland; 2 Department of Materials, University of Oxford, Oxford, OX1 3PH Oxford, UK; 3 Department of Engineering, University of Exeter, Exeter, EX4 4QF UK

**Keywords:** Materials science, Optics and photonics, Electrical and electronic engineering

## Abstract

Neuromorphic hardware that emulates biological computations is a key driver of progress in AI. For example, memristive technologies, including chalcogenide-based in-memory computing concepts, have been employed to dramatically accelerate and increase the efficiency of basic neural operations. However, powerful mechanisms such as reinforcement learning and dendritic computation require more advanced device operations involving multiple interacting signals. Here we show that nano-scaled films of chalcogenide semiconductors can perform such multi-factor in-memory computation where their tunable electronic and optical properties are jointly exploited. We demonstrate that ultrathin photoactive cavities of Ge-doped Selenide can emulate synapses with three-factor neo-Hebbian plasticity and dendrites with shunting inhibition. We apply these properties to solve a maze game through on-device reinforcement learning, as well as to provide a single-neuron solution to linearly inseparable XOR implementation.

## Abstract (web summary)

Some types of machine learning rely on the interaction between multiple signals, which requires new devices for efficient implementation. Here, Sarwat et al demonstrate a memristor that is both optically and electronically active, enabling computational models such as three factor learning.

## Introduction

Efficient neuromorphic sensors and processors have emerged, originally based on CMOS technology^1,2^ and more recently using memristive nanodevices, or memristors^3,4^. The latter are devices that can not only memorize a value in their adjustable physical state, e.g. their conductance, but also can use that value to perform in-memory computations on an externally applied signal, e.g. to emulate synaptic weighting on applied voltage^5–7^. However, more advanced neural mechanisms, such as three-factor learning^8^, dendritic computation^9^, and composite plasticity rules rely on interactions of multiple signals. These mechanisms are fundamental for the optimal learning and processing in the brain, but also lower the computational demands of deep learning^10^, could circumvent current deep learning limitations^11^, and enable functionality that is not possible conventionally^12,13^. Consequently, a neuromorphic device with multi-factor in-memory processing would be highly impactful. A memristive approach that enables the interaction of two distinct signals, such as an electrical and an optical signal, could be a candidate for such a neuromorphic device^14^. Here we demonstrate such an electrical-optical dual-signaling framework, using germanium selenide (GeSe_3_) memristive nano-cavities. We then show advanced neurosynaptic mechanisms emulated by these devices, namely three-factor plasticity and dendritic computation. Ultimately, we demonstrate the application of such mechanisms in reinforcement learning (RL) and in a classification task, so enabling a diverse set of neuromorphic computations.

Reinforcement learning is a category of biological and machine learning that is commonly used to learn rewarding strategies. For example, deep RL has resulted in impressive results for artificial intelligence, such as outperforming humans in the game of Go^15^. The network’s synaptic weights in these algorithms are typically updated based on the interaction between a temporal signal and a reward signal. Preliminary results on RL using memristive synapses do exist but have relied on hybrid digital-analog approaches, where the actual learning aspect is carried out in digital CMOS^16^. A single-device synapse accommodating RL could be realized if the device enabled memory for weight storage but also in-memory three-factor synaptic plasticity^8^. This is a versatile and upcoming category of efficient and biologically plausible learning rules that also enables surprise-based learning^17^, top-down-feedback governed supervised learning^9^ and approximations to backpropagation^10^. From the interaction of multiple signals in biological neurons emerge computations that are not even achievable using standard artificial neurons^12,18,19^ and are not yet exploited by state-of-the-art AI models or their hardware implementations. For instance, the XOR logic gate is a common example of a linearly non-separable problem of classification, that requires multiple layers of conventional artificial neurons for its solution. Nevertheless, it has recently been shown that a single biological neuron can solve this problem using dendrites^12^. Neuronal dendrites are in fact equipped with a variety of powerful computing mechanisms with shunting inhibition being a prime example^20^. Strikingly, this broad family of advanced and desired neuromorphic properties and applications is enabled here by the optomemristors developed in our work. Overall, in this work, we demonstrate multi-factor in-memory computation for reinforcement learning and logic operations. We achieve this by using the tunable electronic and optical properties of Ge-doped Selenide based optical nanocavities.

## Results

### Non-volatile and volatile crossbar optomemristors

**Fig. 1:**
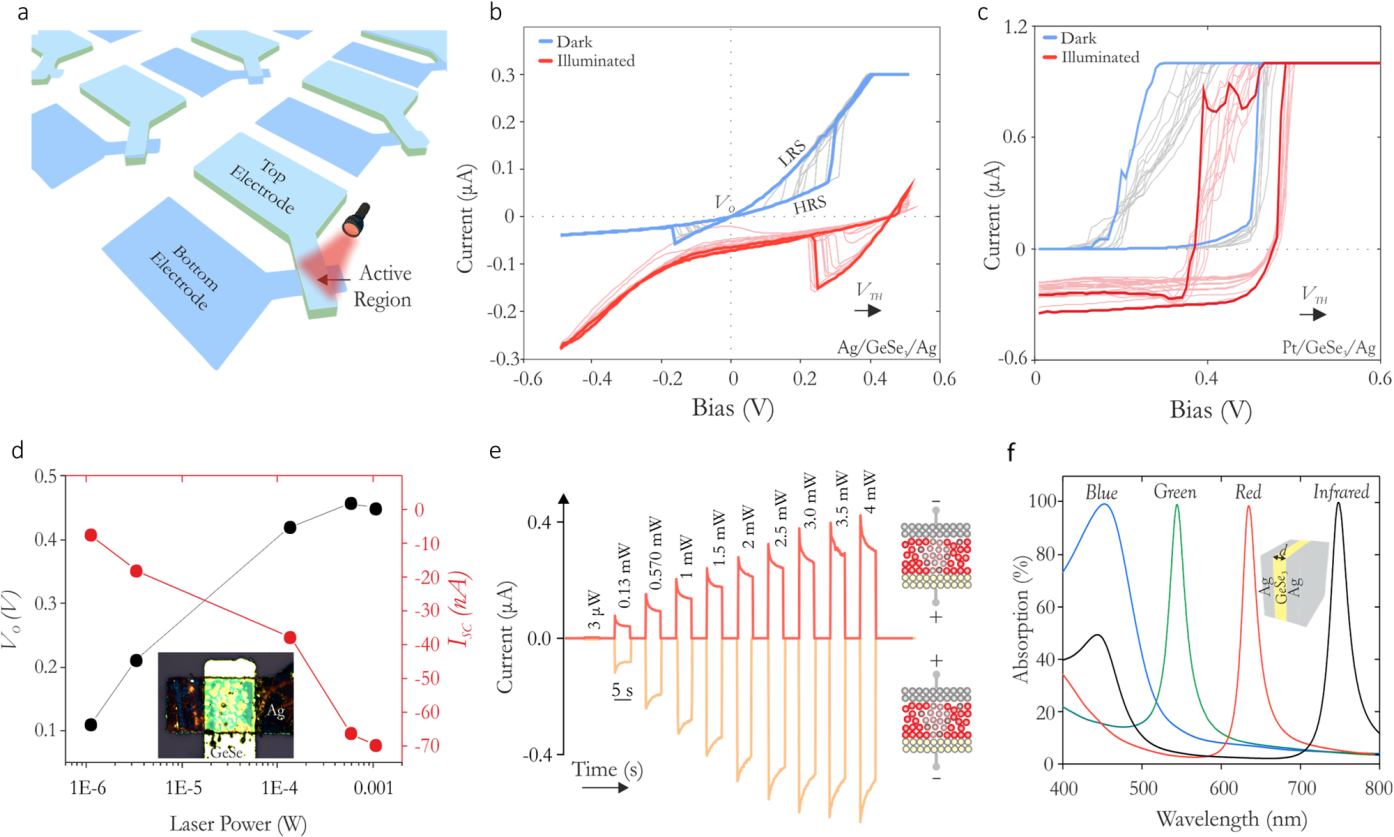
Non-Volatile and Volatile Optomemristor. **A** An illustration of crossbar devices. **B** Current–voltage traces of an Ag/GeSe_3_/Ag non-volatile device under dark and illumination (637 nm/1 mW). Multiple measurements are overlaid in this plot (using a darker color shade a single measurement is highlighted for both conditions). **C** Similar measurements on a Pt/GeSe_3_/Ag volatile type switching device. In **B** and **C** the saturation in current is a measurement artifact due to the compliance current values set during measurements. **D** Cross-over voltage (*V*_0_) and the short circuit current (*I*_sc_) as a function of the illuminating laser power. Inset is an optical micrograph of the device. **E** Short-circuit current in a device as a function of illumination intensity (637 nm) under positive and negative device polarities. **F** The absorption spectra in Ag/GeSe_3_/Ag stacks for blue (*d* = 28 nm), green (*d* = 51 nm), red (*d* = 78 nm), and infrared light (*d* = 103 nm), for varying thicknesses (*d*) of the GeSe_3_ films.

Our devices are solid-state crossbars (Fig. 1A), comprising stacks of thin films of the top (TE) and bottom (BE) electrodes with GeSe_3_ sandwiched in between (see Supplementary Section S1–S2). The electrical resistivity of our device is determined by a conductive channel between the top and bottom electrodes, the formation of which is controlled by an electrical field. However, in our devices, resistive switching can also be controlled optically. This optical responsivity introduces an additional control mechanism. Figure 1B illustrates the current–voltage (*I*–*V*) characteristics of an Ag (BE)/GeSe_3_/Ag (TE) stack under dark (blue trace) and illumination (red trace) conditions. The high resistance state (HRS) of the device indicates an incomplete conductive channel between electrodes, and the low resistance state (LRS) is indicative of an intact and conductive channel, with these two states being stable (i.e. non-volatile (also see Supplementary Fig. S1.1)). However, under optical illumination at 637 nm, the RESET (channel rupture) voltages of our devices shift from the negative bias to the positive bias, i.e. the memristor loses its non-volatility since after SET the device spontaneously RESETs when the applied voltage is removed. The threshold voltage (*V*_TH_) or switching voltage, at which the conductive channel forms in these devices also increases under optical illumination. We find the shift in *V*_TH_ to be notably significant (∼100% relative change) across devices, for modest sub-mW optical power (at 637 nm). When one of the Ag electrodes is replaced with Pt (see Fig. 1C), the device (Pt/GeSe_3_/Ag) spontaneously turns-off (LRS → HRS) when the voltage is ramped below some holding voltage (i.e. becomes volatile). The optical modulation is also observed in such volatile devices, where the device maintains its volatility, while its switching voltage shifts to larger threshold voltages under optical exposure. Notably, because the same active memristive material provides the feasibility to implement both non-volatile and volatile device types – which distinctly enable diverse computational primitives – it eases back-of-the-line integration processes. In what follows, we, therefore, discuss both the non-volatile (Ag/GeSe_3_/Ag) and volatile (Pt (BE)/GeSe_3_/Ag (TE)) devices, and their use case in neuromorphic engineering.

Under increasing illumination, we observe the shift in the switching voltage in both the non-volatile and volatile devices to scale proportionally with the intensity of optical illumination (see Fig. 1D (for Ag/GeSe_3_/Ag) and Supplementary Fig. S1.2). Interestingly, optical illumination not only changes the voltages at which the device switches to a higher conductance state, but also induces a zero-voltage current. This behavior is similar to the functioning of a solar cell and is suggestive of a photovoltaic effect in the devices, which stems from asymmetric Schottky junctions^21^. In Fig. 1D, the shifts in the cross-over point *V*_0_ (open-circuit voltage, where current is zero) and the negative short-circuit current (at zero-voltage) are plotted as a function of illuminating optical intensity. The values scale with optical intensity, with the cross-over point undergoing a shift by 455 mV for an optical intensity of 1 mW. For a device that operates under a photovoltaic mode^21,22^, the direction of the short-circuit changes with the device polarity, which is indeed observed in our devices. This is illustrated in Fig. 1E where the photocurrent (short-circuit current) at zero voltage for a Pt/GeSe_3_/Ag device is plotted as a function of the illuminated optical intensity under differing polarities.

All the layers in our devices are optically thin (the electrodes are typically sub-50 nm thick, the chalcogenide layer typically 20–100 nm thick). These layers behave as resonating optical nano-cavities^23,24^. The cavity design allows us to make devices to selectively interact with different wavelengths, for example from the visible to the infrared, through appropriate layer thickness control. We modeled such effects using transfer matrix calculations with exemplar results shown in Fig. 1F where an Ag/GeSe_3_/Ag device is designed to work the blue, green, red, and infrared regions of the spectrum. Light absorption with high-quality factors and near-unity absorption coefficients are possible by simply varying the thicknesses of the constituting layers.

**Fig. 2:**
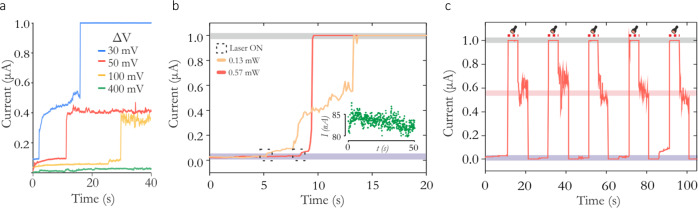
Switching dynamics in volatile Pt/GeSe_3_/Ag devices. **A** Stochastic switching processes under dark conditions. ∆*V* is the difference between the applied constant bias and the device’s intrinsic switching voltage. **B** Light-induced switching under a sub-threshold illumination intensity, and at high optical intensity illumination for bias polarity that produces positive short-circuit current. Inset is the device current under dark conditions. **C** Switching behavior of the device to pulsed illumination. For threshold illumination, the device spontaneously switches to LRS.

When the polarity of either device (Pt and Ag electrodes-based stacks) is reversed, i.e. in a Pt electrode-based device, the Pt pad becomes voltage source, while Ag pad the sink, we find that the conducting channels are still formed. However, with the inverted polarity, optical illumination causes the shift in the switching voltage towards smaller values, i.e. switching occurs at reduced voltages under optical illumination (Supplementary Fig. S1.2). Importantly, these observations show that the devices can be optically controlled to either switch at smaller or at higher voltages relative to their intrinsic switching voltage. We also observed a variable time delay for the onset of switching, with the delay being a function of the applied bias, with voltages closer to the switching voltage increasing the spontaneity of switching (see Figs. 2A and S5.1). However, for electrical conditions under which that device does not switch, we find that optical stimulation can result in switching. In Fig. 2B, for example, we show the response to illumination of a device biased near its switching voltage (in dark conditions). For low-intensity illumination (yellow trace, (0.13 mW), the device undergoes a series of minor switching events before fully switching into the LRS. This effect shows the interplay between long and short-term plasticity, where a high conductance non-volatile state is realized through a series of intermediate states^25^. However, for illumination intensities that can change the switching voltage to below the applied bias voltage (here 0.39 V), the device undergoes spontaneous switching into the LRS (red trace). Such behavior is shown in Fig. 2C, where a pulsed threshold illumination spontaneously switches the device. Once the device is switched, it retains its state (LRS), and the device can then be RESET to the HRS by bringing the applied (bias) voltage to zero. It is clear from the results shown in Figs. 1 and 2 that our GeSe_3_ optomemristor devices offer not only the functionalities generally associated with electrical memristors but also a range of additional, advanced functionalities arising from the combination of electrical and optical means.

### Neuromorphic Computing

#### Three-factor synaptic plasticity

**Fig. 3:**
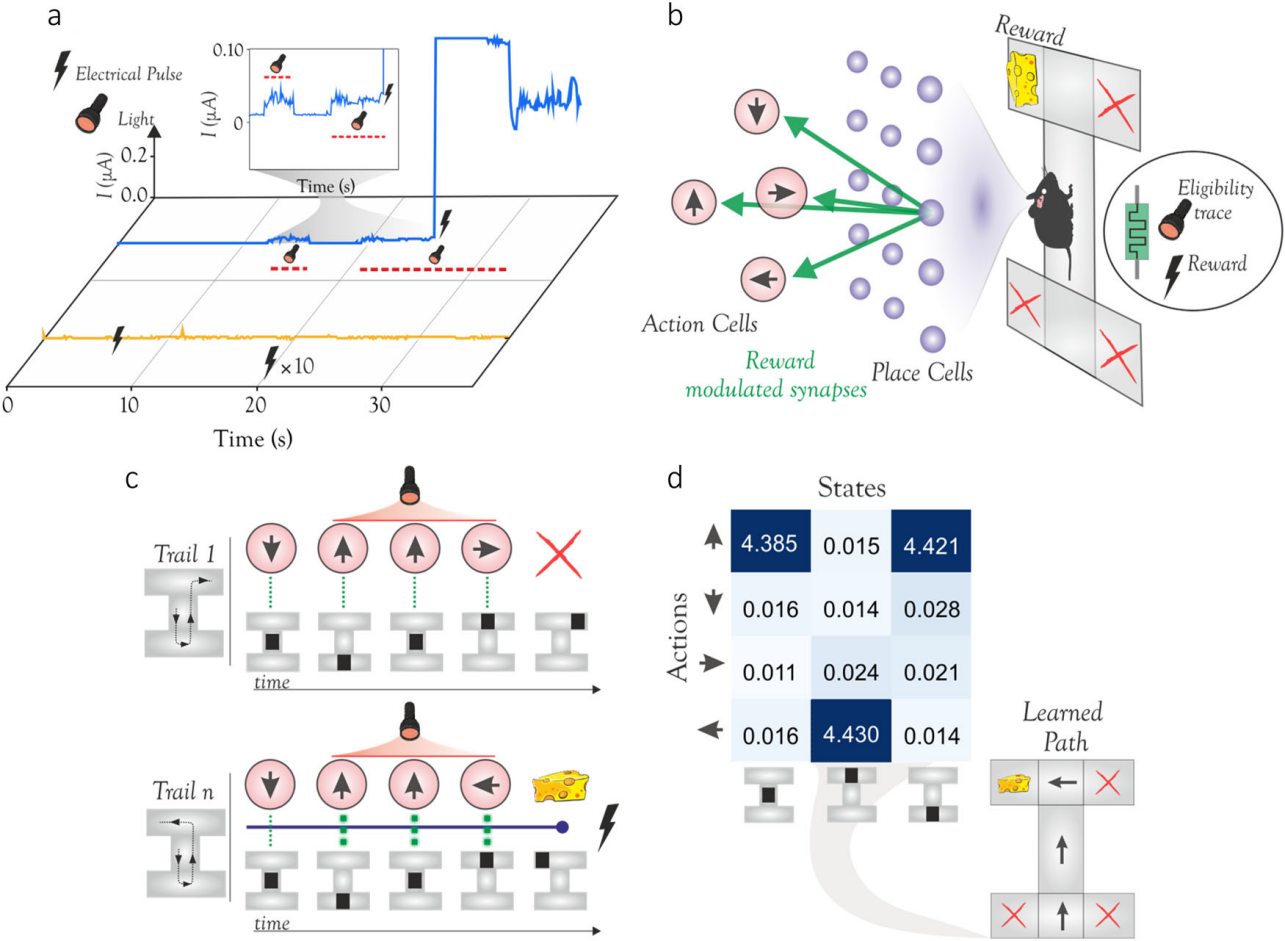
Emulation of three-factor synaptic plasticity and reinforcement learning. **A** Mixed mode behavior of a non-volatile Ag/GeSe_3_/Ag device. In the absence of light (yellow trace), electrical pulses applied to the device do not induce a switching event. Under illumination (blue trace) however, electrical pulses can trigger HRS to LRS switching. **B** Sketch of a rodent in a maze. Place cells represent the rodent’s location in the maze, such that, at each location, one unique place cell is active. Each action cell represents one of four movement directions, and each location triggers one of the four movements. Initially, all synaptic weights equal zero, and through the exploration of the maze and reinforcement learning the rodent learns the weights that enable correct navigation from the initial position to the cheese reward. Each synapse and its weight here are emulated by a non-volatile type memristive device and its conductance. Reinforcement learning emerges through a three-factor synaptic plasticity rule. The rule involves an eligibility flag, which in our case is the illumination of the corresponding memristor, and a reward applied as an electrical signal sent to all memristors. **C** Example trials during the rodent’s training. Each time the rodent moves, an eligibility flag (optical signal) is raised at the synapses of the corresponding place and action cell (red trace). The three eligible synaptic weights are not updated by the optical flag alone, e.g. in an unsuccessful trial (top sequence). A successful trial provides the electrical reward that potentiates the eligible synapses (bottom sequence). **D** Results of training. In the learned weight matrix of the neural network, the electrical conductance (in µS) of the memristive synapses maps each place to an action. This learned mapping corresponds to the correct path to the cheese (inset).

As a first example of the computing capabilities possible using our optomemristor devices, we describe the implementation of three-factor synaptic plasticity, with important applications in reinforcement learning. We utilize the combined effects of the electrical and optical stimulus to modulate the switching behavior of non-volatile Ag/GeSe_3_/Ag type devices. In Fig. 3A, a device is continuously biased at a voltage of 100 mV, below the switching threshold. Electrical pulses that are 500 ns wide (with the rise and decay time of 5 ns) and 400 mV in amplitude are applied to the device under dark conditions. These voltage pulses alone cannot induce switching of the device. Similarly, we also observe that when the device is illuminated but no voltage is applied (blue trace), the device does also not undergo switching (see inset). However, when the device is illuminated and an electrical pulse is then applied, the device spontaneously switches to a conducting state. This is the optomemristive property that we now exploit to deliver a form of neo-Hebbian learning.

Traditionally, computational neuroscience and neuromorphic computing have been focusing on learning algorithms based on Hebbian types of synaptic plasticity^26^. While Hebbian learning can be successful in several unsupervised learning scenarios, and even outperform supervised deep learning schemes in certain cases such as object classification^27^, recent work has shown that other important biological learning tasks, broadly those governing aspects of behavioral learning (e.g. motor control, guidance, and navigation) and synaptic consolidation and tagging require the influence of a third factor, separate from the activations of the two local neurons^8,28,29^. Such neo-Hebbian learning requires three-factor plasticity rules^8,30^ that involve a delayed third signal, such as the reward, as a key factor in the learning process. In AI, such reward-based or RL has been driving some of the most impressive AI successes of the field^15,31^.

Navigation is one important area of learning in which three-factor plasticity and RL is thought to play a key role. For instance, a neuronal cell representing a spatial location of a rodent in a maze, i.e. a place cell, and an action cell representing a particular action that the rodent may take, will strengthen the synaptic connection between them if the rodent takes that action frequently when it finds itself in that location: as a result, the rodent would learn habitually to take that action in that location. This co-activation of the place and action cells is a form of three-factor, neo-Hebbian learning. In this case, the effect manifests itself as a so-called eligibility flag^8,28,30,32^ that makes the pair’s (i.e. action and place neuron) synapse eligible for an update if, and only if, a reward signal is provided within a limited time window. We demonstrate this via an example game where a rodent navigates in a maze to find cheese and avoid traps (see Fig. 3B). A piece of cheese is hidden in one corner of the maze, while the other corners have traps that cause the rodent to be placed back to its starting position. The rodent must learn the appropriate action to associate with each position, which will lead it consistently to the cheese. The action can be one of four (move north, east, south, or west), and the mapping between a place and an action is represented in a neural network, connecting place cells to action cells. For a particular place cell, the most strongly connected action is chosen. The connections, i.e. synapses, are implemented by our optomemristors.

In the implementation of the above maze, we measure the conductances of the HRS and LRS states of individual devices, before, during, and after their electrical and optical illumination. The experimentally extracted conductances are then used as synaptic weights and as updates, in performing the RL simulation on a standard computer. In this simulation, initially, the neural network comprising the synapses is untrained, and all synaptic weights are set to zero., i.e. devices are reset to their low conductance state. The training happens as follows (see Fig. 3C). The rodent explores the maze by taking random actions. Every time an action is taken, the eligibility flag is raised at the corresponding synapse, which in our case is by illuminating the device. This eligibility trace can have arbitrary profiles, such as exponentially decaying or stepped. The stepped waveform is typically used in the synthetic implementations of RL, including in this work^8,33,34^. In our example, we arbitrarily choose the flag to remain raised for three-time steps but allows the expectation for a reward for all possible trials including when the longest path to the cheese is taken in this particular example. If the rodent finds the cheese, an electrical pulse representing a global reward signal is given to all synapses, both the inactivated and those activated during the exploration trial. But, only the last three place-action pairs, if they lead to the reward are potentiated to their high conductance state. Through this process, the rodent successfully learns the correct synaptic weights between the place and action cells that guide the rodent to the cheese in future. In Fig. 3D we plot a quality (Q)-table of the learned network, which maps the favorable actions when in given states through the conductance states of the synapses. For example, when in the maze center, the rodent will preferably move north (up direction), since the corresponding synapse has the highest conductance. Overall, this practical example demonstrates synaptic devices with in-situ three-factor plasticity, and their application in an RL task, made possible by the multi-factorial, i.e. optical and electrical, response properties of these memristors. Compared to other emerging hardware approaches our devices provide an energy efficient and potentially more scalable implementation of three-factor RL^35,36^ (also see Supplementary Fig. S6).

### Shunting inhibition dendrites

**Fig. 4:**
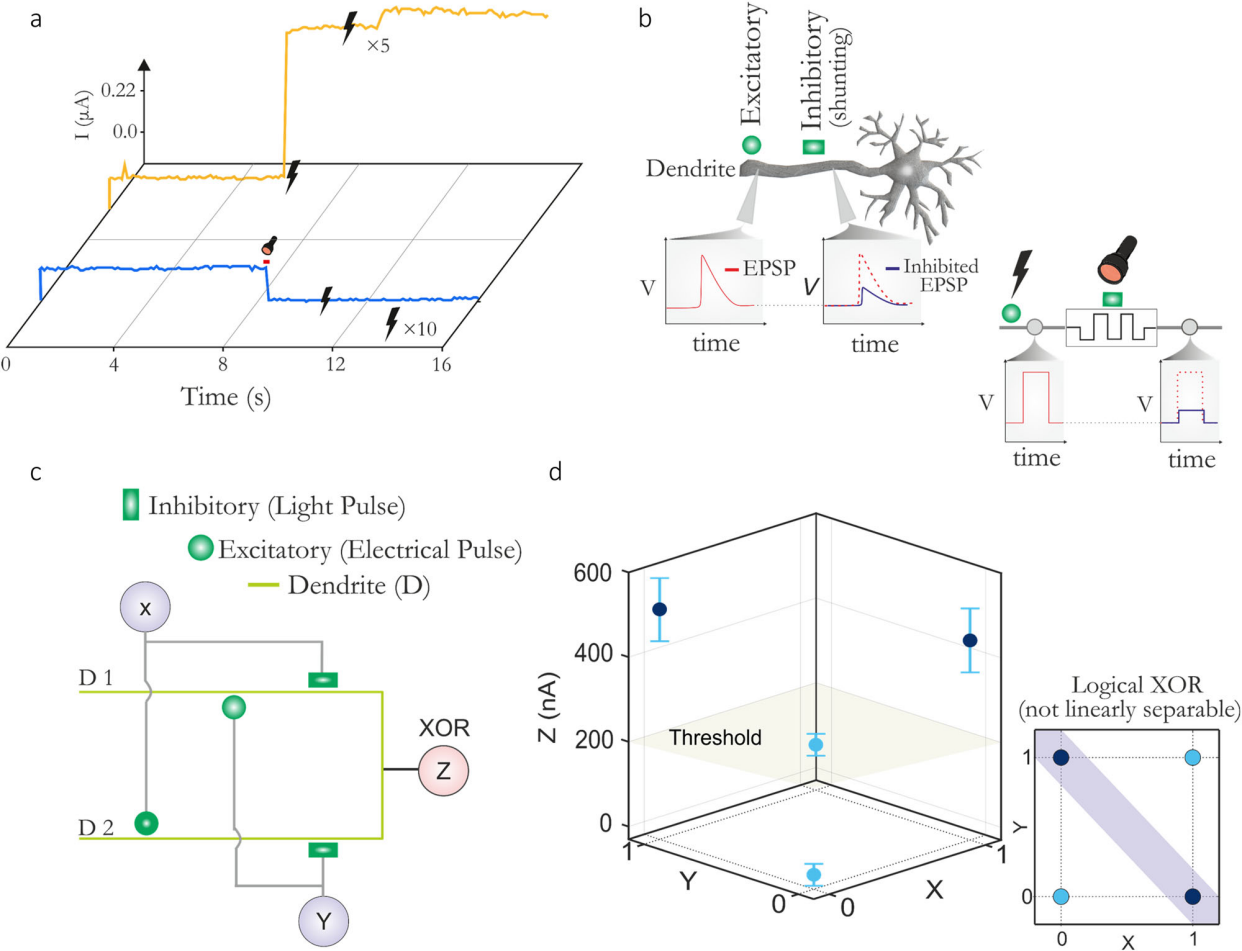
Shunting inhibition and single-neuron implementation of XOR. **A** A volatile Pt/GeSe_3_/Ag device under a mixed mode operation. In the absence of light (yellow trace), electrical pulses applied to the device induce a switching event, while the presence of light inhibits device switching. **B** Sketch of a biological neuron. Shown is a single dendrite of the neuron possessing an excitatory synapse and, proximally to the neuron’s soma, a synapse capable of shunting inhibition. Each excitatory input generates an EPSP (red trace) that propagates, but its effect is gated by inhibitory inputs. An input to the shunting inhibitory synapse attenuates the EPSP (blue trace), but in the absence of excitation, it has no effect (not shown). A volatile type memristive device emulates this dendrite, with the excitatory and shunting inhibitory inputs applied by electrical and optical stimulation respectively. **C** A neuron (Z) comprising two distinct dendrites (D1 and D2) that receive inputs from neurons X and Y. Each dendrite has an excitatory and a shunting input synapse and functionally emulates the biological counterpart shown in **B**. **D** Results of Z’s output for different input pairs. Errorbars represent the mean of multiple measurements. Owing to the memristive dendrites, neuron Z realizes an XOR gate, a function that is impossible for a single layer of point neurons. The green plane defines the activation threshold of Z.

We now demonstrate the second example of single-device multi-factor neuromorphic computation that is enabled by our optomemristors. For this demonstration, we use the volatile devices to emulate single-device dendrites with shunting inhibition, one of the three fundamental types of connectivity between biological neurons^20,37–39^. We then use these shunting dendrites to demonstrate a single-neuron implementation of an XOR logic gate. The XOR is a textbook example of a classification problem that in ANNs requires a network rather than a single neuron for its solution^13,18,19^. Biological neurons however can implement XOR by virtue of nonlinear operations in their dendrites^12^. We here use our optomemristor devices to reproduce this increase in computational power brought about by dendrites. In Fig. 4A, the relevant electro-optical control of a Pt/GeSe_3_/Ag device is illustrated: when illuminated the device’s conductance drops, due to the generation of a negative photocurrent. For these conditions, even when electrical pulses are applied the device is restricted from undergoing filamentary switching. Under dark conditions, however, the device spontaneously switches from HRS to LRS, when an electrical pulse is applied. This is the property that we exploit.

In biology, shunting inhibition describes the following communication mechanism between neurons^20^. Excitatory input to a neuron causes an excitatory postsynaptic potential (EPSP) that propagates through the dendrite. In certain cases, a shunting inhibitory synapse is attached to the same dendrite, but more proximally to the neuron’s soma. If the neuron receives input also from the shunting synapse at the same time as an excitatory input, then the EPSP is shunted, i.e. canceled, or attenuated (see Fig. 4B). Our memristive dendrite receives excitatory input as an electrical signal and shunting inhibitory input as optical illumination. An electrical pulse causes current to pass through the device, but coincident illumination inhibits (i.e. shunts) the current, thus mimicking shunting inhibition (see Fig. 4B). Notably, illumination alone does not increase the device conductance, representing the shunting-only effect of this inhibition. Thus, a single memristor device is able to emulate a dendrite with two adjoining synapses, namely an electrical excitatory one, and an optical inhibitory one.

We then show how a neuron with two such dendritic devices can implement a solution to the XOR (Fig. 4C) ‘problem’. Here the neuron Z receives input from two such memristive dendrites and produces the logic output. Dendrite 1 has two inputs: Neuron X sending a synaptic electrical excitatory input and Neuron Y an inhibitory optical one. On the other hand, for dendrite 2, Neuron Y sends an excitatory electrical input and Neuron X send it the optical inhibitory one. The effects of shunting inhibition on each dendrite ensure that if both X and Y are active, the postsynaptic potentials on both dendrites are attenuated. However, if exclusively X or Y is active, then a postsynaptic potential propagates through one of the two dendrites and activates the neuron Z. The graphical representation of the simulated outputs is illustrated in Fig. 4D. The dark circles highlight the combinations of inputs (X,Y) that activate the output neuron; for other combinations the neuron is inactive. The green XY plane represents the neuron’s threshold, above which the neuron outputs 1. Markedly, the proposed device connectivity realizes an XOR logic gate within a single neuron, by exploiting the added nonlinearity of shunting inhibition at its dendrites. Interestingly, other single-neuron solutions to XOR have been previously hypothesized and considered theoretically possible in biological neurons, contrary to ANNs, due to dendritic nonlinear computations^9,13,40^. One such solution was very recently confirmed experimentally in the human brain^12^. We note that the standard neural net implementation of XOR requires multilayer perceptron with at least ten transistors and six memristors^19,41–43^. Thus, our device represents the increased effectiveness of neuromorphic computing compared to conventional ANNs.

### Device switching mechanisms and discussion

Overall, we explored seven electrode/GeSe_3_ material combinations for optomemristive behavior (see supplementary section S1). Based on the nature of resistive switching (see Supplementary Fig. S1.1) - which was either non-volatile (Ag/GeSe_3_/Ag devices), volatile (Pt/GeSe_3_/Ag devices), or absent (all other combinations) - we infer that the switching process involves a mobile element, namely Ag and that the (non) volatility is dependent on the electrode material; ruling-out switching to involve electron instability and oxygen vacancies, effects commonly reported in Ge rich composition of Se based glasses^44,45^. On lateral devices (Ag/GeSe_3_), we observed that under an applied voltage, the switching event is preceded by the formation of a dendritic structure or the filament (see Supplementary Fig. S1.3). In such devices, the solid-electrolyte was observed to be no longer a uniform thin film structure after sputter-deposition. Instead, it comprised of segregated globule-like structures (likely particles^46^ of Ag) that were uniformly embedded in the surface and volume of the GeSe_3_ matrix. Such nanostructures may exist due to the spontaneity of silver dissolution in chalcogenide glasses^47–50^ (see Supplementary Fig. S1.3–1.6) and were also found to be depleted in the filament’s proximity (see Supplementary Figure S1.5). An elemental spectroscopy map (using energy dispersive X-ray) shows that the filament is rich with constituent elements, including Ag. Furthermore, the molecular make-up of the filament is observed using Raman spectroscopy to be uniquely different from the matrix and bare GeSe_3_ film. Characteristic peaks of the vibrational modes of Se and Ge−Se are quenched near the filament, complementing the EDX profiles in suggesting that the filament is a multi-component structural unit^51,52^, likely Ag_2_Se (see Supplementary Fig. S1.4). We also performed transmission electron microscopy studies. The diffraction patterns showed the GeSe_3_ film matrix to be amorphous, while the filament to be crystalline. As additional proof of filamentation, we tested devices of differing areas. When in the SET state, the filament dominates the resistance of the device; thus while the HRS should decrease with the device area, the LRS is expected to show no scaling, which is precisely what we observe (see Supplementary Figure S1.7). These observations combined with the optoelectronic measurements which showed an increased charge collection (larger photocurrent) in the LRS states of devices indicate filamentary behavior as the primary mechanism to describe the observed switching effects. The observed dependency of filament’s stability (volatility) on the electrode material is likely dictated by minimization of the interfacial energies between the filamenting material, the dielectric, and the electrode, effects that have been observed in other memristors^53^. The observations so far, collectively suggest that the mode of optical tunability in the devices studied here is photovoltaic (see Supplementary Fig. S2.3) and governed not only by simple electromigration of a conductive filament from a host electrode but also by the electric field-driven re-arrangement and precipitation of the already dissolved electrically conductive nanostructures at the electrodes. Such effects have been shown to induce polarity independent filamentation in other material systems, including Ag/ZrO_2_, Ag/TaO_5_, Ag/ZnS stacks^53–55^. We note that the optical absorption in GeSe_3_ for the light frequency we use is only modest (see Supplementary Figs. S2.4 and S3.1), and that, for higher optical powers/absorption, photo-driven phase-segregation of Se could limit the operability of the devices. Thus, GeSe_3_ is a good demonstrator system, while other materials, such as ternary compositions and tellurides^51,56^ may be better-suited for applications that will make use of the optical modulation in the visible and infrared. We also note that existing work has demonstrated the usefulness of the GeSe-based optically controlled memristors^14,57^, and products utilizing such optical programmability are already commercially available^58^. The cavity design and downscaling of devices shown in this work thus expand the utility of this emerging device concept for computing applications.

We wish to point out that in the specific RL demonstration that we provide, our solution does not address all open challenges of three-factor plasticity for RL. It does arguably provide one important missing piece and proposes how the remaining challenge can be circumvented. Specifically, the temporal aspect and synaptic memory of the trace is but one of the two key missing pieces. The other piece of three-factor plasticity is the interaction between the three factors, within a single device using the mixed-mode approach. Our choice to use light as eligibility traces, and electrical signals for a reward was motivated by the easiness of operation and proof-of-principle demonstrations, however, vice versa can be also adopted. Some important challenges, however, should be pointed out when considering scaling up such an approach. While the use of light for eligibility traces could provide an energy-efficient implementation compared to electrical eligibility traces, from reduced power dissipation in resistive wirings of the crossbars-, it may prove impractical to achieve the locality or uniqueness of eligibility trace signals at the scale of individual synapses in a large-scale network. This is owing to the physical limit of optical exposure area placed by diffraction. This, however, could potentially be overcome using electrical eligibility traces. For example, this would be achieved using electrostatic signals applied to the gate terminal of the devices; a method that would exploit yet another characteristic property of chalcogenide^59^ materials, which is their semi-conductivity. The use of optical eligibility traces may therefore fare well for population encoding of synapses. We did not make use of every possible functionality in our practical demonstrations; however, we discuss a few in Supplementary Fig. S6.

In summary, we have described a framework using GeSe_3_ devices with silver ions acting as a memristive element that is both electrically and optically active. Such an “optomemristor” is shown to be configurable as both volatile and non-volatile, governed by the choice of the electrodes. Our extensive characterization of these electrodes indicates that it is the movement of Ag ions in the GeSe_3_ matrix that enables these effects. We then characterized the unique optoelectronic features of these devices and exploited such features to provide a range of neuromorphic functionalities that rely on multi-signal interactions, such as three-factor plasticity and dendritic computation. We demonstrated two proof-of-concept applications, in-memory RL and multilayer neural computation using a single neuron. The growing success of deep RL and multilayer neural networks, in general, suggests that such neuromorphic optomemristor devices could have a key role in future AI hardware accelerators. All in all, these devices expand the use cases for memristors in computing applications, while providing a vehicle for testing models from computational neuroscience.

## Methods

### Device fabrication

Films were sputter-deposited directly on thermally grown 300 nm SiO_2_ wafers (from IDB Technology, UK). Substrates were first cleaned for 10–15 mins in acetone under ultrasonic agitation, rinsed in isopropanol, and dried with pressurized nitrogen. The bottom electrode of the cross-bar devices was then patterned using standard photolithography (positive resist-S1813: exposed for 14 s, baked at 120 °C, and developed for 45 s in MF319 developer). Reactive ion etching was carried out to embed the electrodes in the oxide. Ta (16 nm) was deposited as an adhesive layer in a Nordiko sputtering system: working pressure of 9.6 µTorr, 44.5 sccm (standard cubic centimeters per minute) Ar, and 120 W RF. Bottom electrode was then subsequently deposited in the same sputtering system: with a typical working pressure of 3.5 µTorr, 11.5 sccm Ar, and 40 W RF, without breaking the vacuum. Following lift-off in acetone with mild ultrasonic agitation, the top electrodes were patterned using the same photolithography procedure. GeSe_3_ deposition was then carried out from a solid target (Testbourne, UK): working pressure of 3.5 µTorr, 11.5 sccm Ar, and 30 W RF. Without breaking the vacuum, top was then sputter-deposited (Testbourne, UK): at unique sputtering conditions. Lift-off was carried out in acetone: 65 °C for 8 h. For nano-gap devices, graphene was patterned using electron beam lithography and the nano-gaps were produced using feedback-controlled electroburning. A self-alignment approach described in Nano Letters 2017, 17, 6, 3688–3693 was used for deposition of GST. We used atomic force microscopy in the non-contact mode to measure the film and the stack thicknesses. Note that our EDX and microanalysis results, both on the chalcogenide sputtering target and the deposited thin film suggest GeSe_3_ as the composition. However, following reference Journal of Physics and Chemistry of Solids 68, 866–872 (2007), the Raman spectra of the films suggest a rather higher concentration of Se, giving a composition close to Ge_15_Se_85_. However, in our experiments (see supporting information Supplementary section S3 and *Nano Lett. 2019, 19, 10, 7377–7384)*, we found that optical exposure during laser exposure in the Raman measurements can induce structural changes to the films. Therefore for purpose of clarity, we choose to represent our film with the composition GeSe_3_.

### Electrical and optical characterization

Electrical measurements were carried out using a Keithely 2614 B sourcemeter, Tektronix AFG000C pulse generator, and Teledyne Lecroy WaveSurfer Oscilloscope. The devices were illuminated using a custom-built probe station with a Gaussian beam spot size of 20 µm for 637 nm laser. Fiber-coupled lasers were used (Thorlabs) for illumination. The devices were wire-bonded using Al/Si wires to a custom-built printed circuit board, which in turn was connected to the measuring units using 50Ω coaxial and SMA cables. All measurements were computerized using custom-built LabVIEW codes. Reflectivity measurements were performed on a custom-built microscope setup. The reflection spectra were simulated using the transfer matrix method, adopted on custom-built MATLAB codes. The refractive index data of the for simulations were experimentally derived using a J.A.Woollam ellipsometer, whereas for Ta and Pt using the existing literature. For the simulations described in the main-text, experimental data on the device conductance under, during, and after optical and electrical pulsing was extracted from a few cells on the same chip. In the optoelectronic measurements the optical illumination was always performed from the top, such that light traverses from the top electrode toward the BE. Illumination itself is performed using a laser beam to avoid stray exposure. Further details are provided in the supporting information.

## Supplementary information

Supplementary Information

## Data Availability

The data presented and used in this publication is available from the corresponding authors on reasonable request.

## Code availability

The code presented and used in this publication is available from the corresponding authors on reasonable request.
