# Supplementary material for: Chalcogenide optomemristors for multi-factor neuromorphic computation
Source: Nat Commun. 2022 Apr 26;13:2247. doi: 10.1038/s41467-022-29870-9 (PMC9042832; doi:10.1038/s41467-022-29870-9)
Supplement: Supplementary file 1 — Supplementary Information [file 41467_2022_29870_MOESM1_ESM.pdf]

Supporting Information

Chalcogenide optomemristors for multi-factor neuromorphic computation

Syed Ghazi Sarwat, Timoleon Moraitis, C David Wright and Harish Bhaskaran

Supplementary Section S1

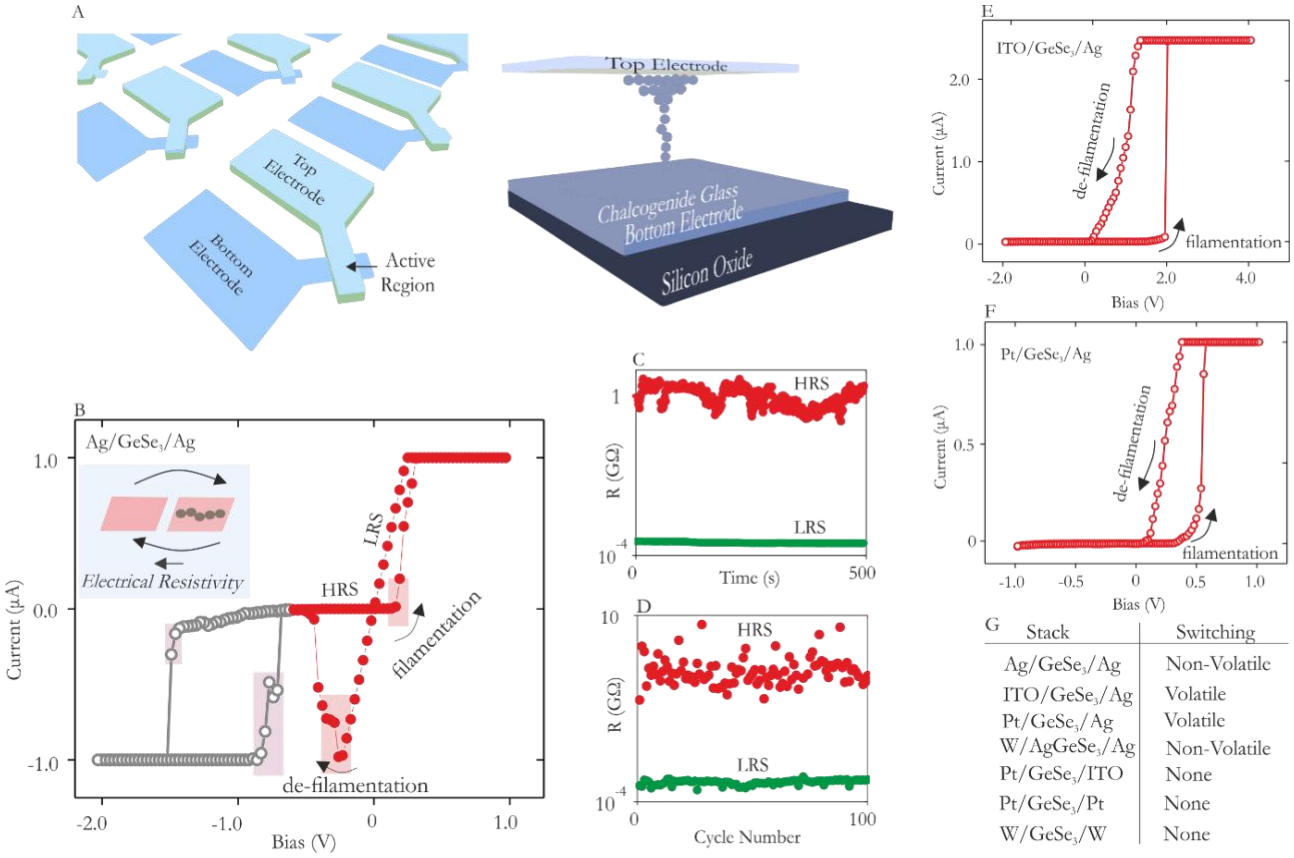

Supplementary Figure S1.1. (A) Schematic of a cross-bar device consisting of a chalcogenide glass sandwiched between conductive electrodes. The electrical resistivity of the device is dictated by a conductive channel illustrated in the inset. (B) Current-voltage characteristics of an Ag (15nm)/insulating $GeSe_3$ (36 nm)/Ag (60nm) device. Resistive switching involves the processes of channel formation (filamentation) and rupture (de-filamentation). The black data points represent switching in the reverse polarity due to the symmetricity of the device. (C and D) Memory endurance test showing excellent separation between on and off states, with no degradation after several switching cycles. (E and F) Current-voltage characteristics of an $ITO/GeSe_3/Ag$ and $Pt/GeSe_3/Ag$ device, respectively. The device undergoes volatile switching, which involves spontaneous de-filamentation. (G) A list of different devices tested and their switching behaviors.

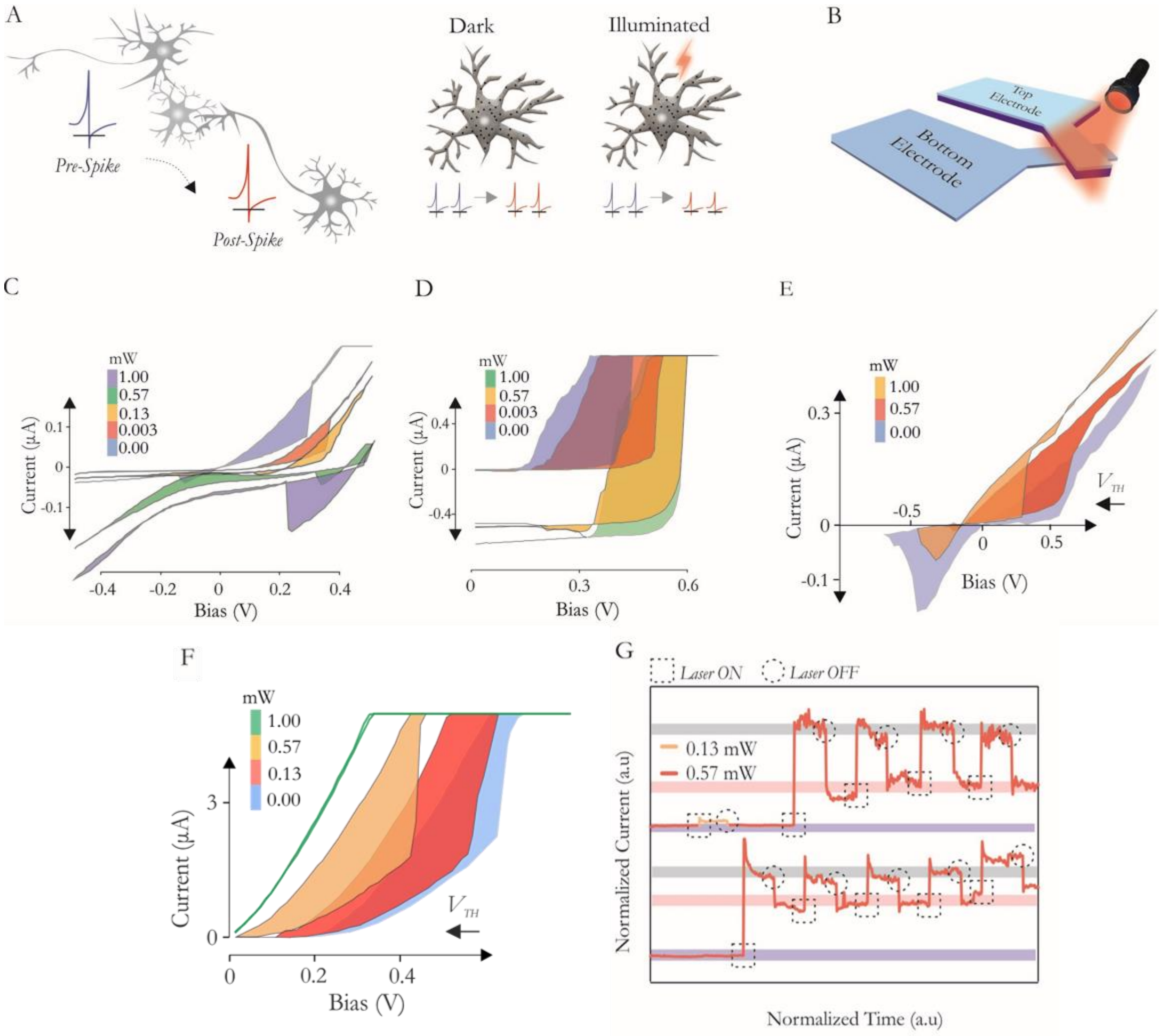

Supplementary Figure S1.2. (A) Structure of interconnected neurons. In the emerging field of optogenetics, nerve cells are deliberately modified to express light-sensitive ion channels, in enabling an ‘optical’ means of commanding learning and memory. In such biological nerve cells, the electrically defined neuronal activity (shown as current spikes) is modulated via optical exposure of gated ion channels (black spots). Crucially, the ability to artificially replicate this unique and important feature of the nerve cells will require memristors to exhibit tunability in their switching dynamics. (B) Cartoon sketch of chalcogenide crossbar. (C and D) Multiple current-voltage traces of a non-volatile $Ag/GeSe_3/Ag$ and volatile $Pt/GeSe_3/Ag$ device increasing illumination conditions. Note the zero-bias (short-circuit) negative photocurrent and the shift in the switching voltage (V_{TH}) under illumination. Under illumination also observe how the RESET voltages in the $Ag/GeSe_3/Ag$ are now placed in the positive polarity. Thus when the applied voltage is removed after SET the device spontaneously RESETs. (E) Switching behavior of an $Ag/GeSe_3/Ag$ under reverse polarity. Note the positive photocurrent and that the V_{TH} decreases with increasing illumination. It is unclear why the V_{TH} shift changes direction when the pads (signal and ground) are swapped since even without electrically we sweep the voltages in both polarities. One possible reasoning could be that once a filament is formed in our bidirectional devices in a certain polarity, the reverse polarity will always ensure a RESET. (F) Switching behavior of a $Pt/GeSe_3/Ag$ under reverse polarity. Note that like before the V_{TH} decreases with increasing illumination. (G) Switching behavior of a $Pt/GeSe_3/Ag$ device to pulsed optical exposure. For sub-threshold illumination, the device produces only a transient photocurrent, while at higher intensities, it spontaneously switches into LRS with greater photocurrent.

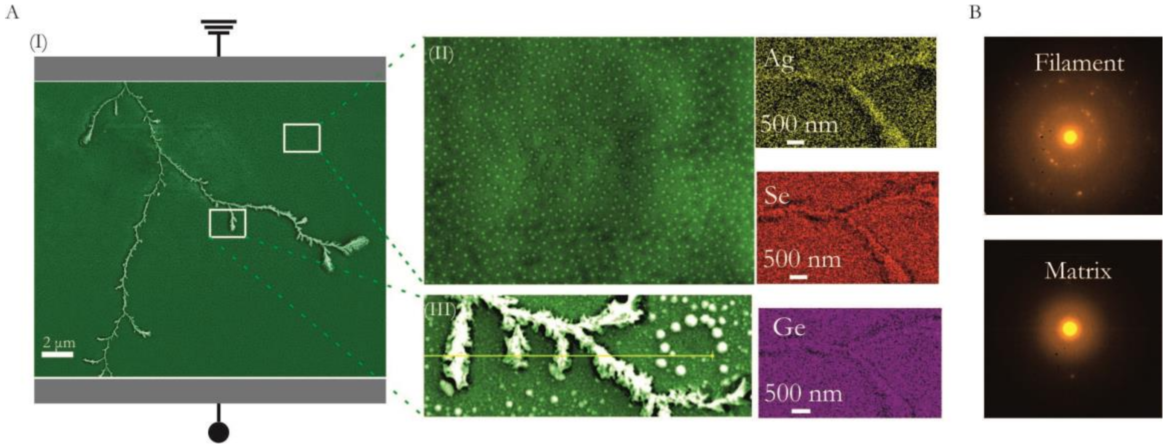

Supplementary Figure S1.3. (A) (i) False-colored SEM micrograph of an $Ag/GeSe_3$ stack. Under an applied electric field using tungsten probes, the device undergoes non-volatile switching, which is preceded by the formation of a dendritic filament. (ii) Inset shows a composite like the structure of the stack wherein nanostructures are embedded in the $GeSe_3$ film. (iii) The bottom inset shows the zoomed-in region of a filament; the yellow line is the elemental scanning line. The right panel is EDX elemental maps of the different elements constituting the device, illustrating the filament to be rich in Ag, Ge, and Se. (B) Electron diffraction patterns of the filament and matrix, showing that the filament is crystalline and the matrix is amorphous.

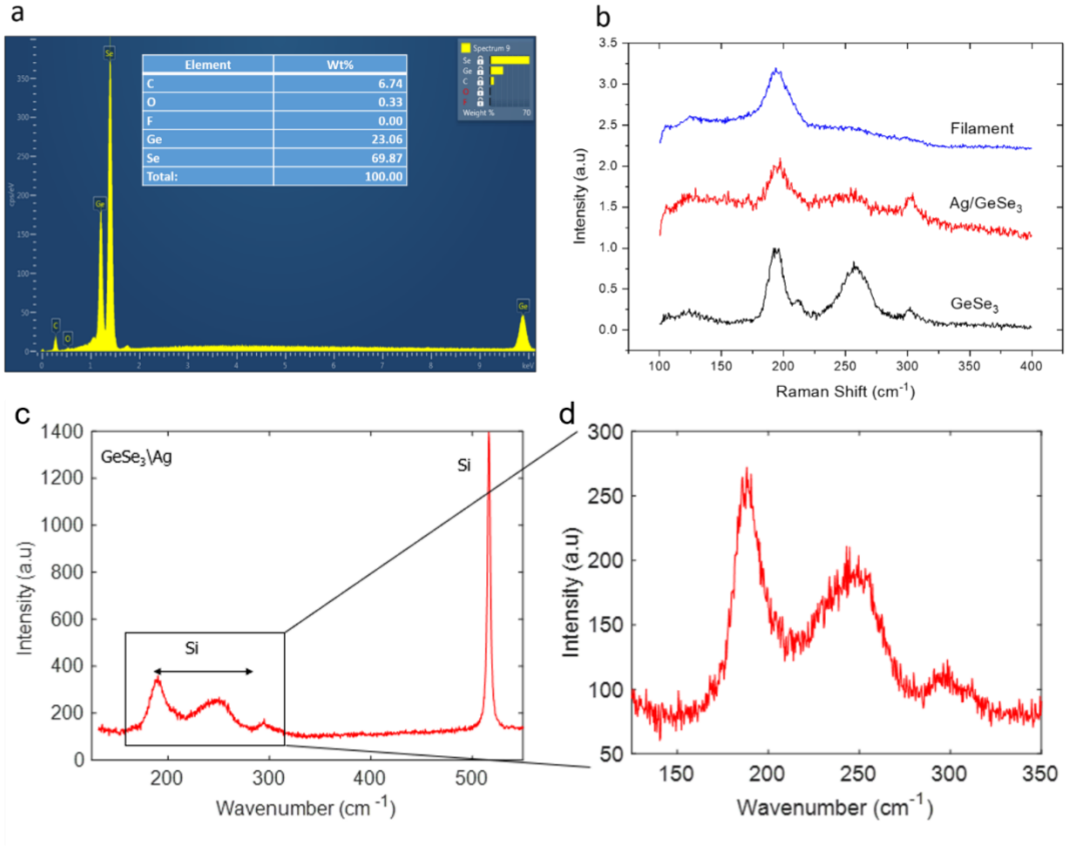

Supplementary Figure S1.4. (a) Energy-dispersive X-ray spectra of a $GeSe_3$ thin film. (B) Raman spectra of a $GeSe_3$ thin film, $Ag/GeSe_3$ stack and conductive filament. The 194cm^{-1} is the ETH vibration modes of the corner-sharing $GeSe_{4/2}$ tetrahedrons and the 211cm^{-1} peak represents the ES breathing vibrations of the edge-shared $Ge_2Se_{8/2}$bi-tetrahedrons. The peak at 262cm^{-1} is the Se–Se bonds and indicates the configuration of Se is in the form of Se-8 rings. At the filament, the ES, Se-Se and Ge-Ge peaks are

suppressed; highlighting a change in the local bonding configuration of the atoms. This observation is in line with previous reports and are suggestive of depletion of Se chains in the matrix via photoactivated reactions between Ag and Se (Journal of Physics and Chemistry of Solids 68, 866–872 (2007)). Note that the Raman measurements were carried out using 639 nm optical excitation and at low optical power (1 mW/3s integration time and 3 accumulation cycles) to avoid photo-induced changes. (c-d) Raman spectra of $GeSe_3$/Ag 36 months after deposition. We would like to point out that our EDX and microanalysis results, both on the chalcogenide sputtering target and the deposited thin fim suggest $GeSe_3$ as the composition. However, following reference Journal of Physics and Chemistry of Solids 68, 866–872 (2007), the Raman spectra of the films suggest a rather higher concentration of Se, giving a composition close to $Ge_{15}Se_{85}$. However, in our experiments, we have found that optical exposure during laser exposure in the Raman measurements can induce structural changes to the films. For purpose of clarity, we choose to represent our film with the composition $GeSe_3$. It is in the interest of our future report to more carefully study the film composition with other analysis methods.

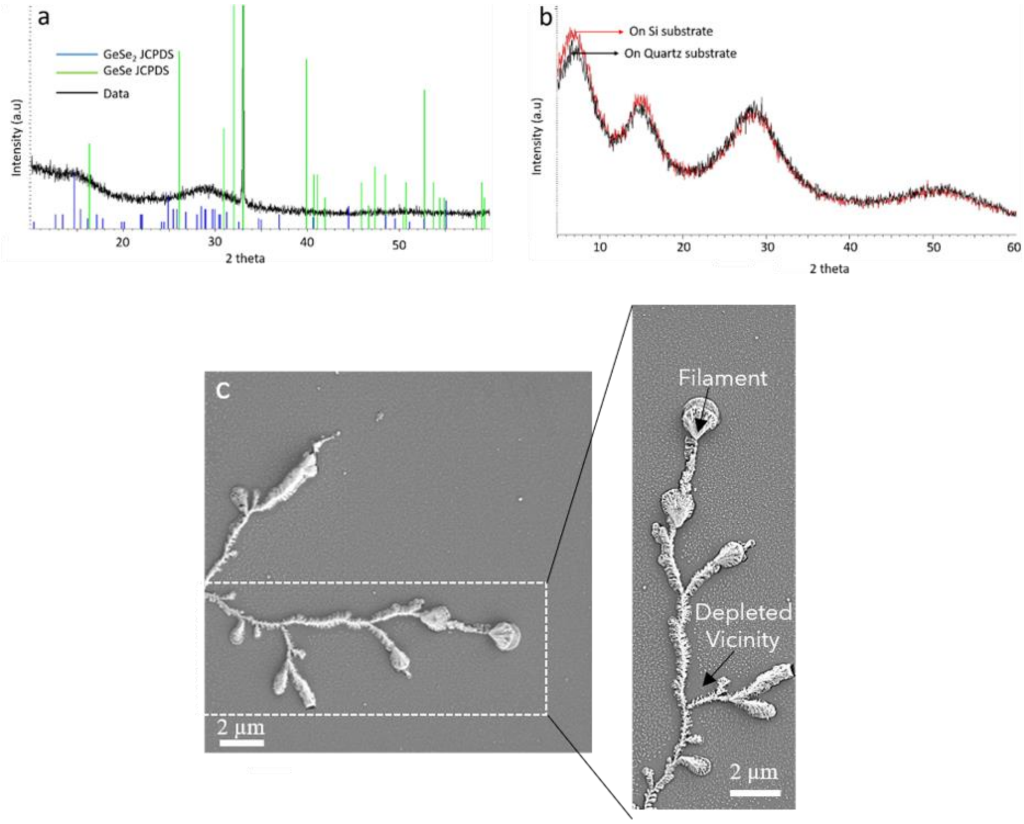

Supplementary Figure S1.5. (a) X-ray diffraction of spectra (black trace) of a 100 nm $GeSe_3$ thin film. The film is amorphous with no characteristic peaks. (b) X-ray diffraction of spectra of a $1\mu m$ $GeSe_3$ thin film at a grazing angle annealed at 330^0C for 6 mins in room conditions. No peak corresponding to crystallites is observed. (c) A scanning electron micrograph of the filament in an $Ag(15nm)/GeSe_3(38nm)$ stack. Inset is a zoomed-in view of the bottom branch. Note that around the periphery of the filament, the matrix is devoid of the nanostructures, which are otherwise homogeneously distributed in the matrix.

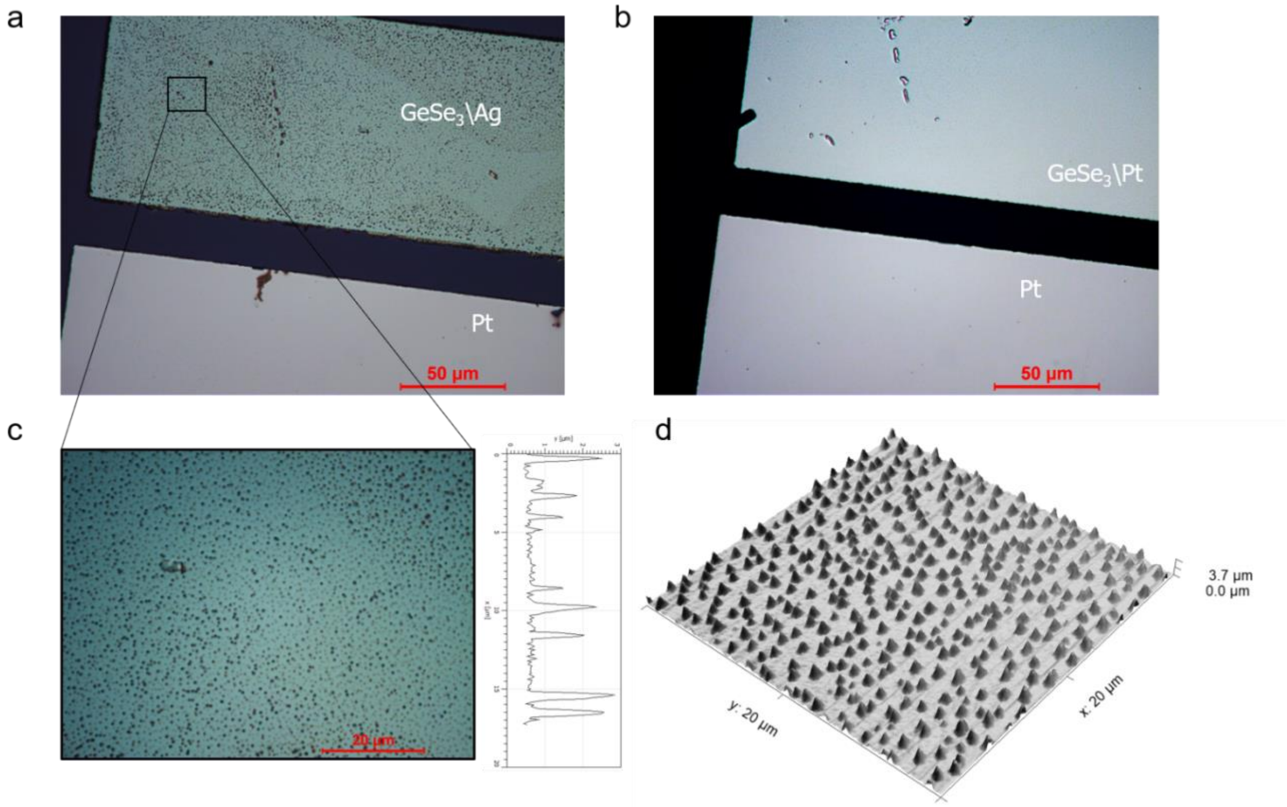

Supplementary Figure S1.6. An optical micrograph of a Pt/$GeSe_3$/Ag device after 36 months from deposition. (b) An optical micrograph of a Pt/$GeSe_3$/Pt device after 36 months from deposition. (c) It is noted that Ag diffuses into the chalcogenide film (which acts asolid-electrolyte), and forms into embedded globules like structure. Pt however is not noted to diffuse. Inset shows an atomic force microscopy line scan on $GeSe_3$/Ag pad. (d) An atomic force microscopy map of (c) showing photo-diffused Ag rich nanostructures. Ag diffuses into the film through photo-chemical processes (Journal of non-crystalline solids 124,186–193 (1990), Journal of Physics and Chemistry of Solids 68, 866–872 (2007), Thin solid films 449, 248–253 (2004), Advances in Physics40, 625–684 (1991)), the extent to which is governed by the saturation limit of Ag doping, which inturnis governed by chalcodgenide electrolyte film (Journal of Physics and Chemistry of Solids 68, 866–872 (2007)).

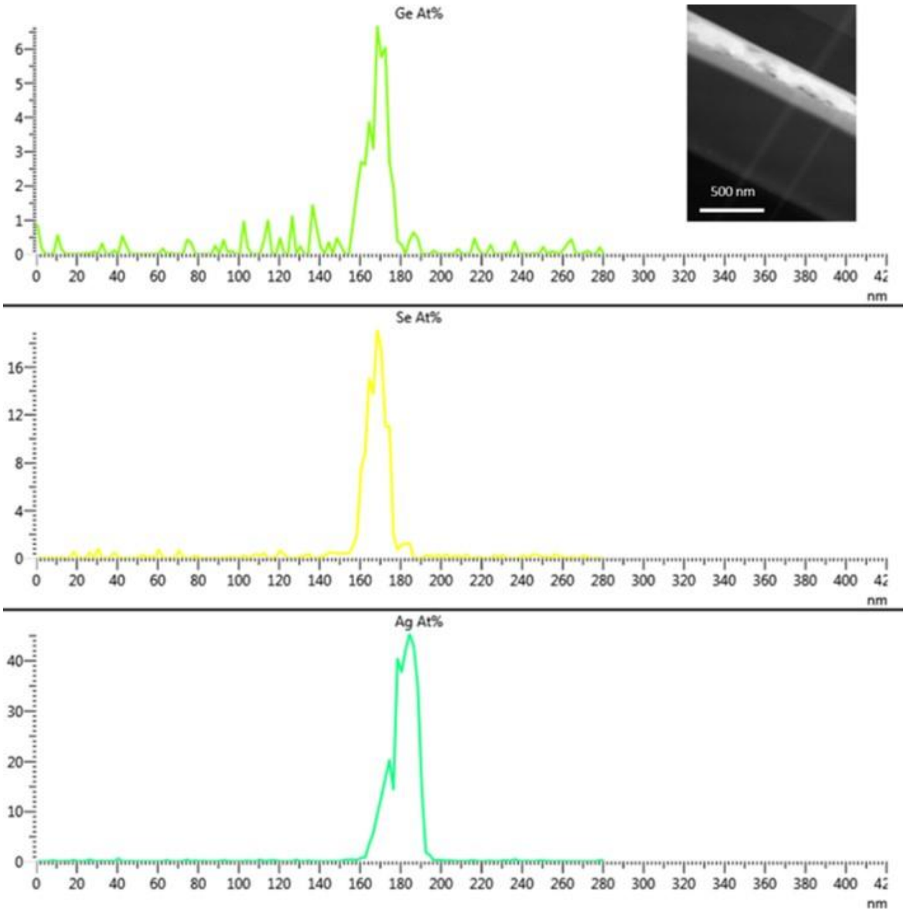

Supplementary Figure S1.7. Energy-dispersive X-ray spectroscopy on an *Ag/GeSe*$_3$*/Ag* stack (top to bottom panels) using transmission electron microscopy operated in the STEM mode. The entire top Ag layer and a major portion of the *GeSe*$_3$ film are sputtered away during focussed ion beam based sample preparation (evidenced by the Ga content in the films). However, the more interesting region is the interface between the bottom Ag and *GeSe*$_3$, which highlights the diffusion of Ag into the *GeSe*$_3$ film. This is a result of the spontaneous intermixing of Ag into *GeSe*$_3$ volume.

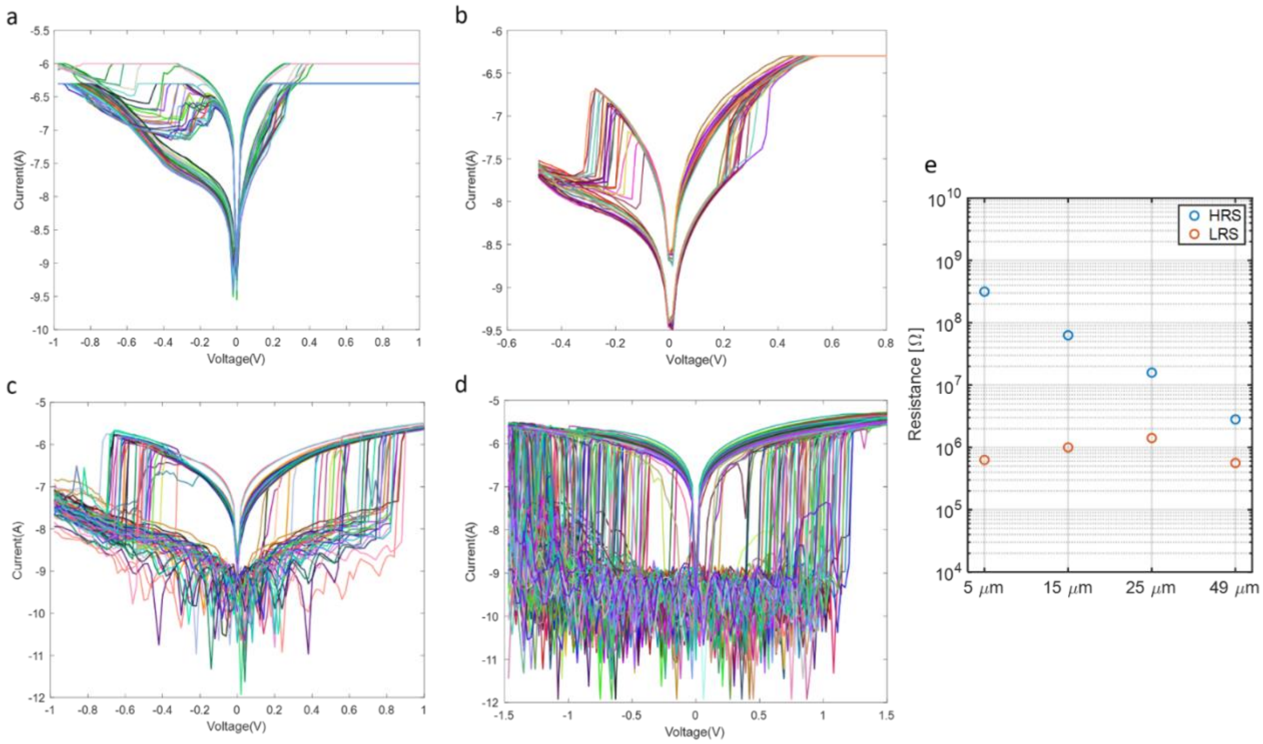

Supplementary Figure S1.8. Cyclic current (10^y)-voltage characteristics of $Ag/GeSe_3/Ag$ stacks of cross-sectional area (a) 49.5μm × 49.2μm, (c) 25.3μm × 24.8μm, (d) 15.2μm × 14.5μm, (d) 5μm × 4.8μm. (e) Resistance vs device cross-sectional area of the devices in both their HRS and LRS states. In a filamentary memristor, the LRS state can be regarded as a parallel configuration of the conductive filament and the rest of the device. Thus, when the device area is changed (the bottom and top electrodes), the resistance of the HRS state (devoid of a filament) is expected to decrease from the inverse relationship of resistance with the area, but the resistance of the LRS state is expected to not change since the dimensions of the filament across which the voltage drop do not change.

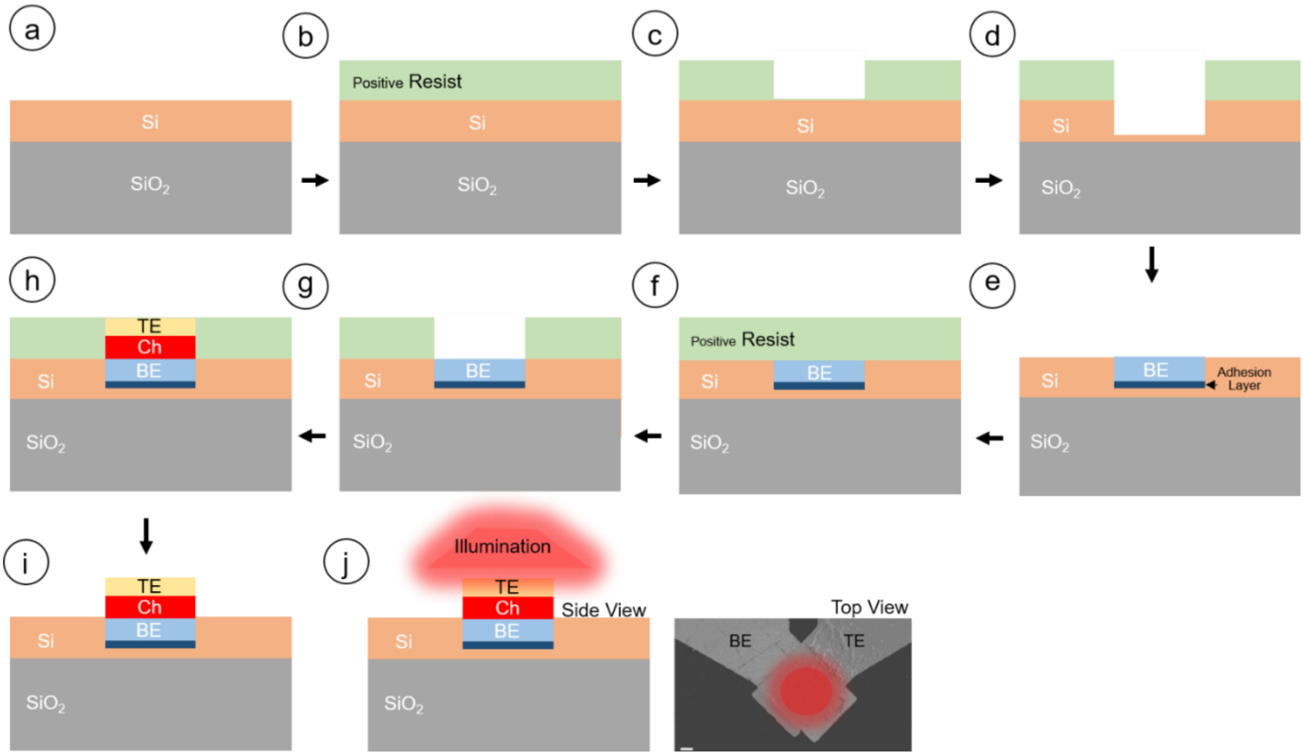

Supplementary Figure S1.8. A 300 nm thin SiO_2 is thermally grown on an n-Si (100) wafer using wet oxidation at 1050 ^{0}C. (b-c) The wafer is coated with an S1813 positive resist for patterning the bottom electrodes (BE) using photolithography (2 mins baking at 120 ^{0}C, 8-20 seconds exposure time/30-60 seconds, development in MIBK developer/30-60 seconds reaction termination in isopropanol). (d) After pattering, the SiO_2 on the wafer is reactive ion etched in the areas exposed by UV exposure (to take the shape of the BE) using the chemistry CHF_3=50 sccm, Ar=10 sccm, O_2=2 sccm, Power=100 W (at etching rate 21 nm/min). The etch depth is carefully optimized to match the BE thickness so that BE is planarized with respect to the SiO_2 surface. (e) Based on the stack type and using RF sputtering the BE is deposited in Ar atmosphere at a working pressure of 3.6e-3 mtorr. 2-3 nm of sputter-deposited Ta was used as an adhesion layer between BE and SiO_2. Sputter conditions are: Ag (Power=30W/ rate=4.5 nm/min) or Pt (Power=40W/ rate = 4.2 nm/min) or ITO (Power=30W/ rate = 3.5 nm/min) and Ta (Power=120W/ rate = 4.6 nm/min). Following this, the metals were lifted off in an acetone solution, placed in a water bath at 65 ^{0}C. (f) Second photolithography using S1813 positive resist is then performed (2 mins baking at 120 ^{0}C, 8-20 seconds exposure time/30-60 seconds) to pattern the top electrode (TE) and chalcogenide $GeSe_3$ (Ch). Global alignment markers are used to align the TE with BE. (g-h) After development (in MIBK developer/30-60 seconds reaction termination in isopropanol), $GeSe_3$ film and TE (Ag, ITO, Pt) are sputter deposited. Sputter conditions for $GeSe_3$ are Power=30W/ rate = 4.9 nm/min under working pressure of 3.6e-3 mtorr. (i) The depositions were then lifted off in an acetone solution, placed in a water bath at 65 ^{0}C, and the wafers were then used for testing. An important fabrication step we note is the requirement to not overfill the trench with BE. If the BE is overfilled and post chemical mechanical polishing is not carried out, then BE and TE are noted to be electrically shorted, making devices electrically conductive and unusable. (j) In our experiments the optical illumination is always performed from the top, such that light traverses from the top electrode toward the BE. Illumination itself is performed using a laser beam to avoid stray exposure.

Supplementary Section S2

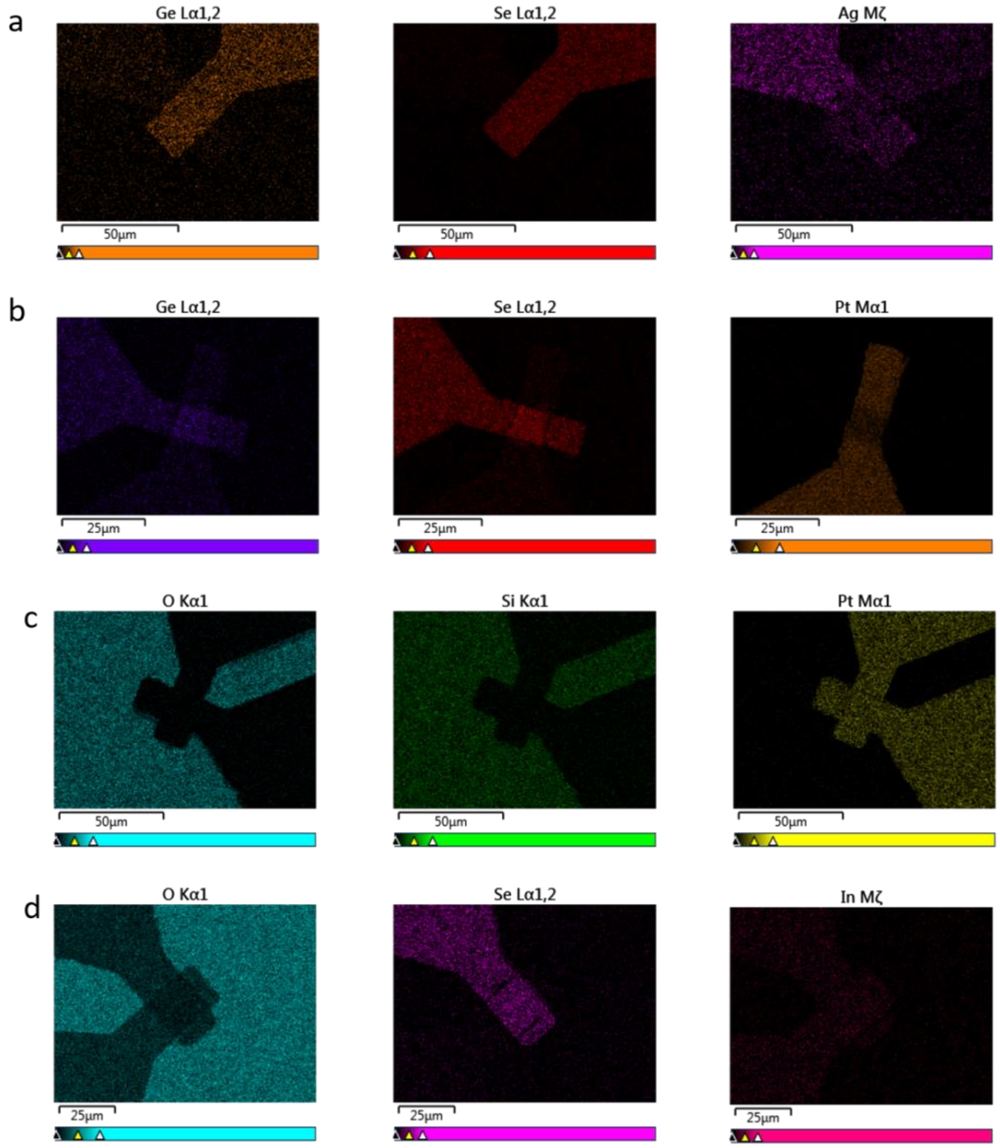

Supplementary Figure S2.1. Energy dispersive X-ray maps of cross-bar devices. (a) *Ag/GeSe*$_3$*/Ag*, (b) *Pt/GeSe*$_3$*/Ag*, (c) *Pt/GeSe*$_3$*/Pt*, and (d) *ITO/GeSe*$_3$*/Ag*.

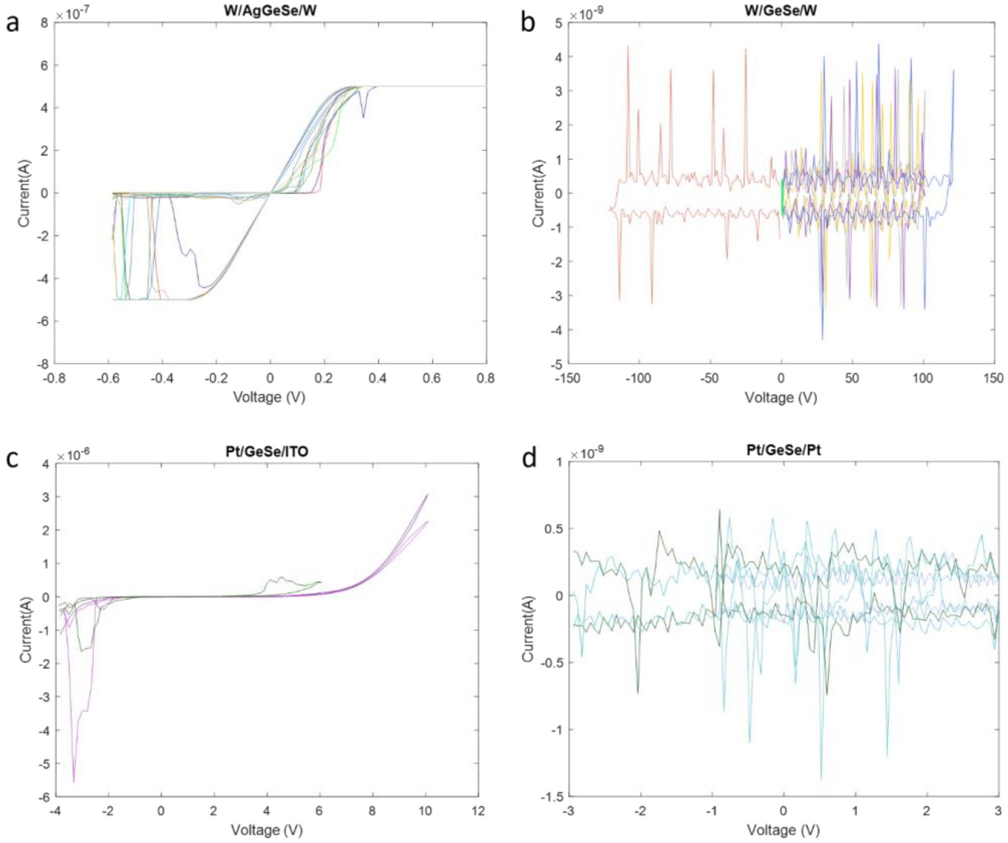

Supplementary Figure S2.2. (a) Current-voltage characteristics of cross-bar devices. (a) *W/AgGeSe*$_3$*/W* stacks, (b) *W/GeSe*$_3$*/W* stacks, (c) *Pt/GeSe*$_3$*/ITO* stacks and (d) *Pt/GeSe*$_3$*/Pt* stacks.

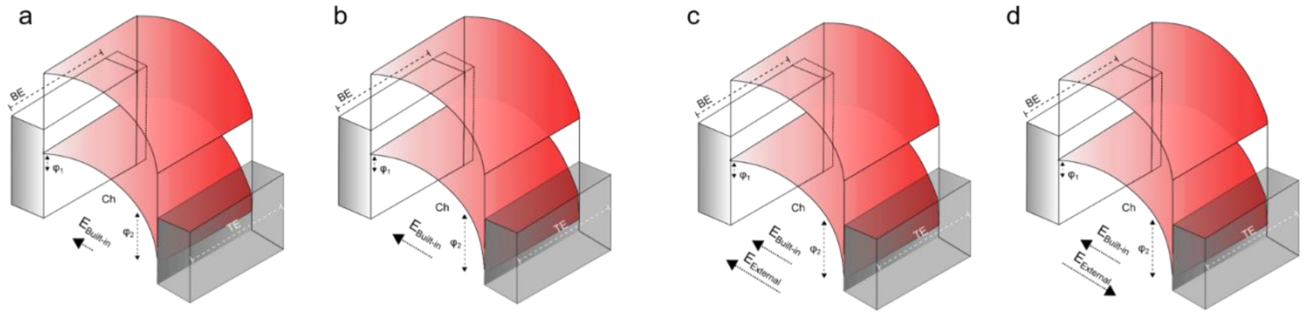

Supplementary Figure S2.3. Illustration optomemrisitive mechanism using energy-band diagrams. (a) The observation of a short-circuit current indicates that our devices work with a photovoltaic mode of operation, governed by asymmetric Schottky barriers between $GeSe_3$ layer and the bottom and top electrodes. This in turn implies that an in-built electric field ($E_{in\text{-}built}$) exists in the $GeSe_3$ layer. (b) Under increasing optical illumination we also find that the open-circuit voltage shifts, which further suggests that the Schottky barriers -thus the in-built electric field- change under illumination. (c) When an external voltage is applied to the channel, an external electric field ($E_{xternal}$) is set in the $GeSe_3$ layer. If E_{TH} is the threshold electric field required to switch the device from HRS to LRS, then the voltage (V_{TH}) required is reduced if both the in-built and external field point towards the favorable filamentation direction. (d) When $E_{in\text{-}built}$ and $E_{xternal}$ are directed in the opposite directions the switching voltage is expected to be larger. Sketches (c) and (d) point to our experimental observations of increasing and decreasing (V_{TH}) for different external voltage polarities. We however note that while our device functions in a photovoltaic mode, the photovoltaic mode does not exclusively render the observed optomemristive switching characteristics. Chalcogenide glasses are a good solid electrolyte for Ag.

Furthermore, a unique feature of chalcogenide glasses Ge_xS_y and Ge_xSe_y is their accelerated diffusion for Ag under the action of light (Journal of non-crystalline solids 124,186–193 (1990), Journal of Physics and Chemistry of Solids 68, 866–872 (2007), Thin solid films 449, 248–253 (2004)). It is understood that under illumination an electrical potential is created via photochemical action, and the resultant electric field provides sufficient energy for Ag cations to diffuse from the interface into the bulk of the chalcogenide film (Thin solid films 449, 248–253 (2004)). Thus, in effect illumination provides an additional knob (beyond electrical voltage) to modify the filamentation dynamics of the devices. Note that photodoping/photodiffusion occurs readily (at a slower rate) in our devices when they are exposed to ambient light. We discussed this in Figure S1.3. In Figure S1.6 we show photodiffusion in our films after 36 months from fabrication. Also note that optical heating inside the active area of the device can modify the equilibrium potentials, aiding the photo-diffusion effects. However, estimating the temperature rise in the chalcogenide film in the active area of the device is non-trivial since this requires an estimation of the optical properties and distribution of the photo-diffused and formed crystal in the active region of the device. We also find that for the used programming conditions, the conductance of the filaments in the LRS state does not exhibit an integer of the conductance quantum, which is likely due to scattering and imperfect contacts (Physical Review Letters 92, 106804 (2004), Nanoscale research letters10, 1–30 (2015)). Therefore, the conductivity in our devices is likely because of quantum mechanical tunneling (Nano Letters16, 709–714 (2016)).

We have simulated optical heating effects in our devices using COMSOL® Multiphysics for estimating the temperature rise in the films. Our model consists of two parts: a 2-D approximation that models the layers of different materials that formed the nano-device. By simulating the way an incident electromagnetic wave of the same wavelength as the laser beam propagated through the geometry, it is possible to quantify the energy losses due to absorption within the device. The second part of the model is a 3-D heat transfer simulation that represented the geometry of the crossbar nano-device. The heat source utilized was calculated based on the energy losses obtained from the first part, therefore assuming that the energy lost due to optical absorption was transformed into heat and dissipated through the device geometry. We however note that the extinction coefficient of $GeSe_3$ for the investigated wavelength is not significant. In Figure S2.4a we plot the absorption in $GeSe_3$ when configured for different stack types. Note that $GeSe_3$ minimally absorbs the incident electromagnetic wave (see Figure S2.4b). These results, however, assume that $GeSe_3$ and Ag are physically separate layers with a sharp $GeSe_3$/Ag interface. In real world, we have observed Ag diffusion into $GeSe_3$, which should alter optical heating in the layer. To account for this, we perform our simulations for higher absorption (see Figure S2.4d(i)) in $GeSe_3$. Figure S2.4b shows the 3D geometry of our crossbar device model. The temperature distribution across the cross-bar structure, obtained by applying 1 mW (same as in experiments) of optical power to the active region of the crossbar is shown in Figure S2.4d(ii). Figure S2.4e plots the cross-section of Figure S2.4d(ii): the interfaces between $GeSe_3$ and Ag electrodes are in the Z direction. In Figure S2.4e(i) it can be seen that a thermal gradient is formed within the device due to heat loss via the Ag electrodes. For Ag we use thermal conductivity value of 429 W/mK and for $GeSe_3$ 0.53 W/mK. Note that the top electrode is optically heated which diminishes its role as an effective thermal sink and the bottom electrode is more effective in dissapting heat. The central region of the device reaches the peak temperature, but it is clear that the temperature rise in the chalclgenide film is minimal owing to the effective thermal sink provided by the Ag electrodes and only modest optical absorption in $GeSe_3$. In Figure S2.4e(ii-iv) we repeat our simulations for a larger incident optical powers (5 mW, 10 mW, 65 W), which indicate the increase in the peak temperature due to greater optical heating. We however note these models need further experimental validation, and it is in the interest of future work to collaborate experimental and simulations findings on different chalcogenides systems to more exclusively delineate the optical heating and photochemical effects in the memristive switching behavior of devices.

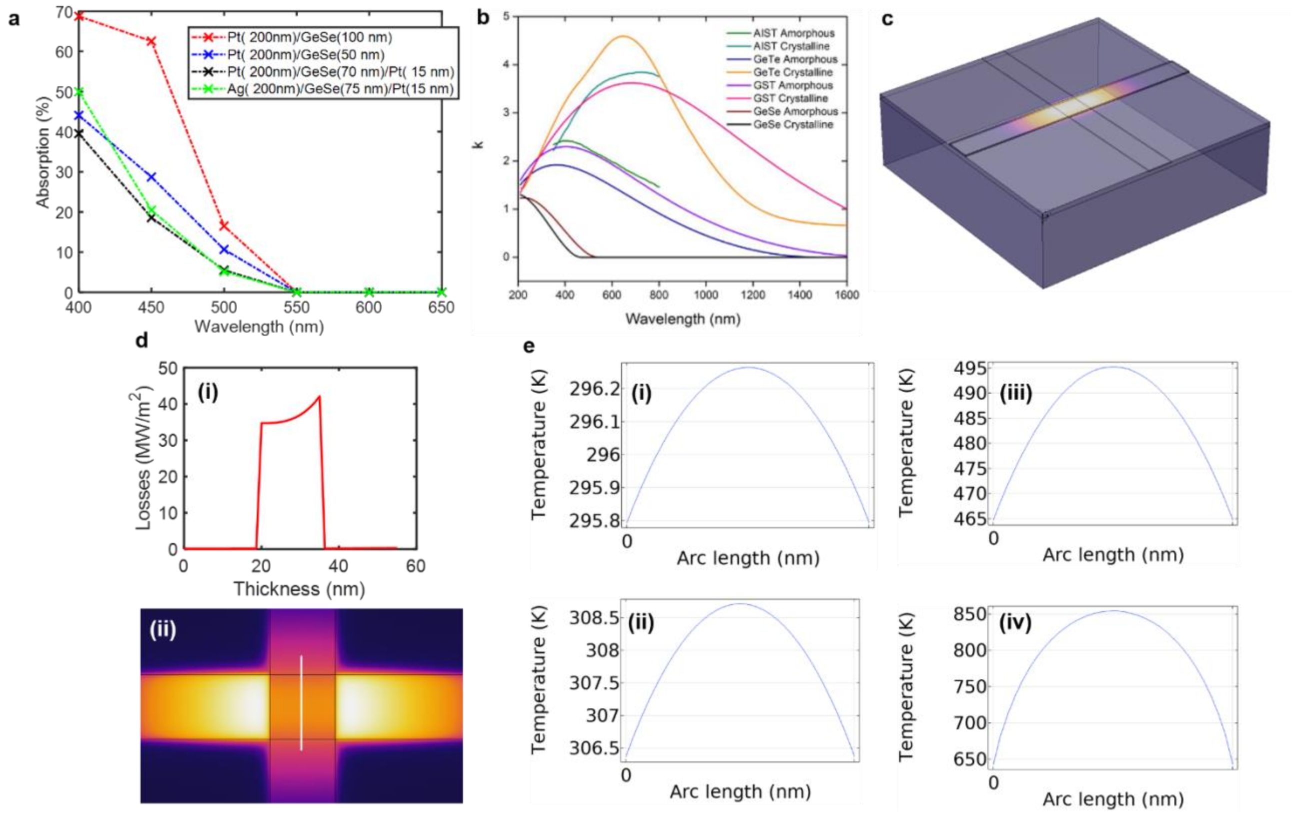

Supplementary Figure S2.4. (a) Absorption in $GeSe_3$ film when configured into different optical cavities. (b) Optical extinction coefficient of a few chalcogenide systems. $GeSe_3$ is most transmissive for the investigated wavelength (633 nm) among the listed systems. (c) An illustration of a 3D heat transfer model of a Ag(20nm)/GeSe(15nm)/Ag(20 nm) crossbar . (d) (i) An approximated electric field distribution of a normal incident beam on the stack. (ii) A 2D heat map showing optical heating in the device, that scales with the input laser power. (e) Cross-sections of the temperature gradient along the line inset in c (ii), for (i) 1 mW, (ii) 5 mW, (iii) 10 mW and (iv) 65 mW input laser power. Note that in our experimental demonstrations the laser power was limited to ≤1 mW.

Supplementary Section S3

Optical Cavity Simulation: Optical simulations were performed using a transfer matrix approach. The approach is analogous to a scattering matrix that relates the initial state and the final state of a physical system (electric fields) undergoing a scattering process. The refractive indices required for estimating the Frensel coefficients in this approach were obtained experimentally using ellipsometry.

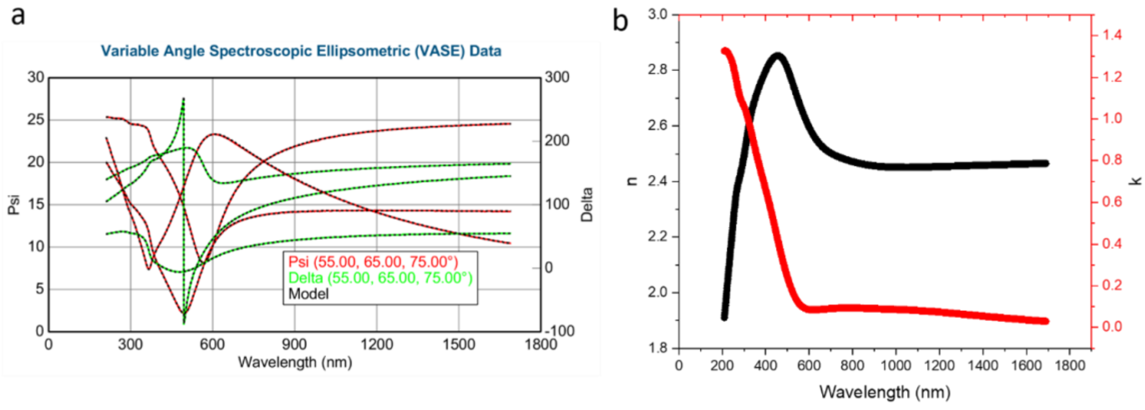

Supplementary Figure S3.1. Optical properties of a (6nm) Ag/(30 nm) $GeSe_3$ stack. (a) Raw elliposmetry data (Psi) fitted with a B-Spline model. The model fits adequately with the data. (b) Refractive index of the stack. For further reading on constructing optical nanocavities with $GeSe_3$ see *Nano Lett. 2019, 19, 10, 7377–7384*.

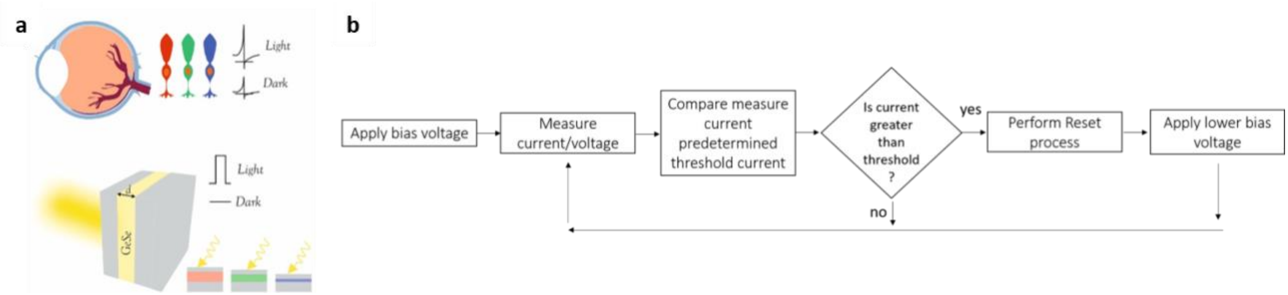

Supplementary Figure S3.2. (a) Structure of a human eye. Insets are the dedicated photoreceptors for red, green and blue light, which trigger an action potential for photon detection. The bottom panel is the color tunability achievable in filamentary devices by optimization of thicknesses of the layers in the stack: red (Ag(100 nm)/$GeSe_3$(78 nm)/Ag(35 nm)), green (Ag(100 nm)/$GeSe_3$51 nm)/Ag(35 nm)), blue (Ag(100 nm)/$GeSe_3$(28 nm)/Ag(15 nm)) and infrared (Ag(100 nm)/$GeSe_3$(103 nm)/Ag(35 nm)) light. (b) A proposed scheme for a spiking photodetector utilizing the light modulated filamentary devices, for applications such as the artificial retina. Photon (s) detection occur by a high resistance to low resistance switching event in the device. The devices can be reset to their high resistance state at the clock frequency after every spiking event; by applying negative or zero voltage pulses for non-volatile and volatile devices, respectively.

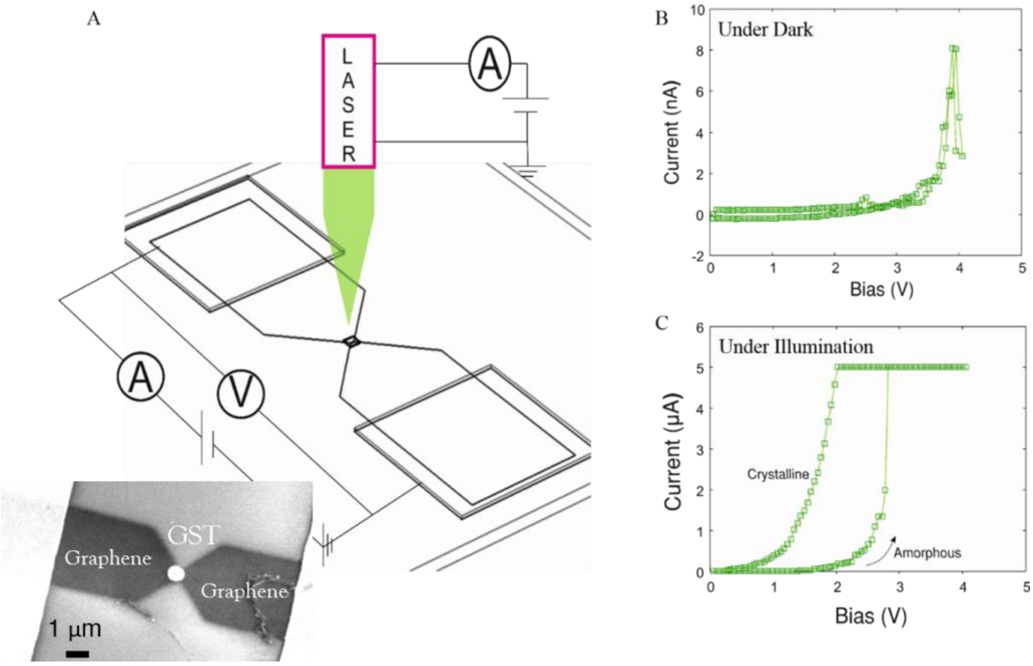

Supplementary Figure S3.3. Optical control of switching in a phase Change Material ($Ge_2Sb_2Te_5$). (a) A schematic of a graphene nano-gap device ($Ge_2Sb_2Te_5$ island bridges a ≈ 20 nano-gap) and apparatus for measurement. Inset is a SEM micrograph of a typical nanogap device. (b) Current-voltage characteristics of the detector measured in the dark conditions. (c) The current-voltage characteristics of the detector measured under illuminated conditions. Note the device switches from amorphous to crystalline in a non-volatile manner highlighted by the hysteresis in the current. The truncation at $5\mu A$ is the compliance that the measurement unit is set to.

Supplementary Section S4

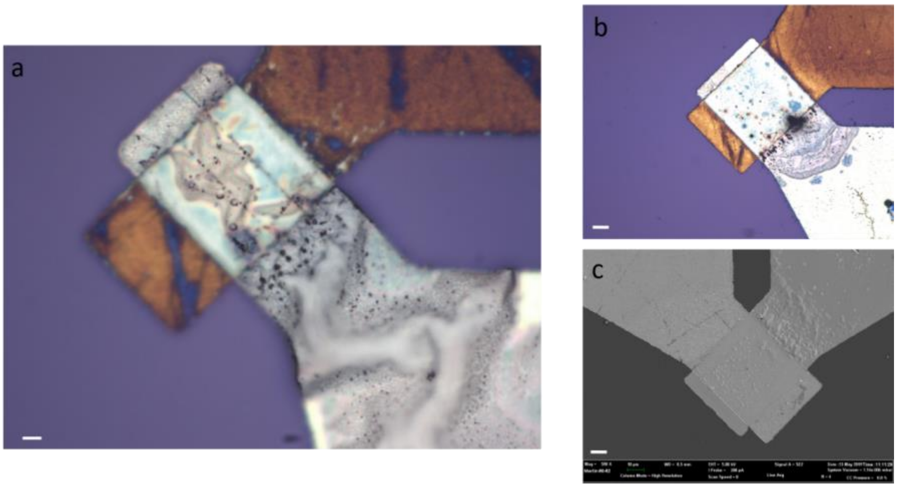

Supplementary Figure S4.1. (a) An optical micrograph of a cross-bar device after high-intensity optical exposure (5 mW/637 nm). The greenish region is a result of photo-induced changes in the $GeSe_3$ film, and the wrinkles are likely due to the delamination of the film. (B and C) An optical and scanning electron micrograph of a different device showing the structural change and device damage from optical exposure. The scale bar is $10\mu m$.

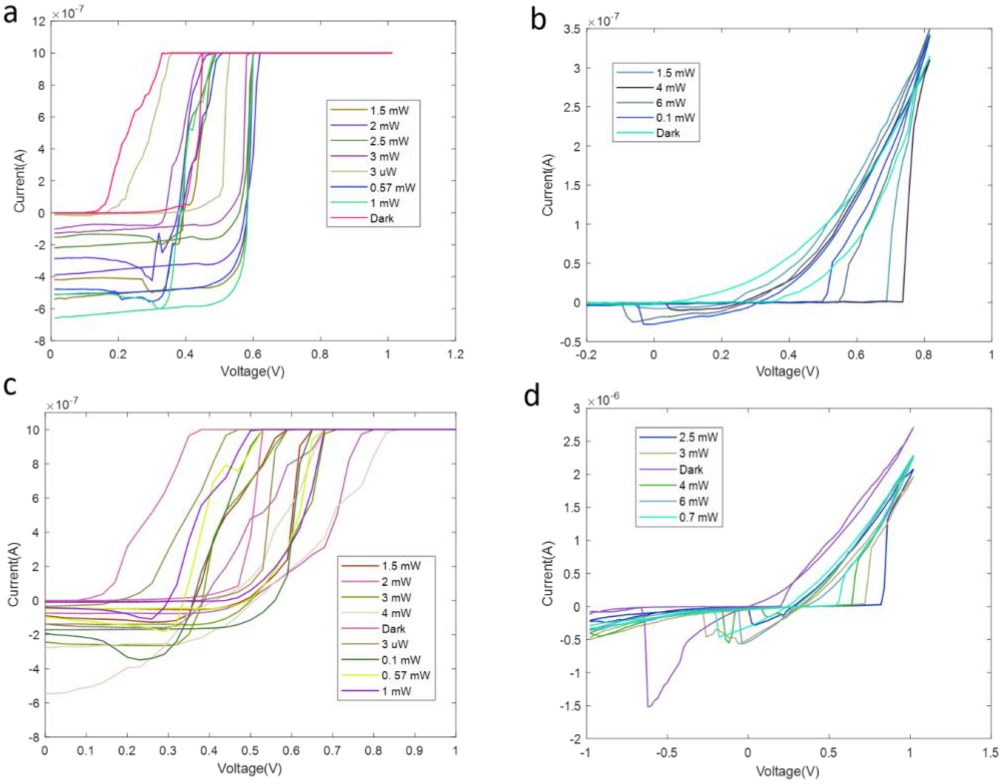

Supplementary Figure S4.2. Current-voltage characteristics of more cross-bar devices under higher intensity illumination conditions. (a) A *Pt/*$GeSe_3$*/Ag* cross-bar device. (b) An *Ag/*$GeSe_3$*/Ag* cross-bar device. (c) Another *Pt/*$GeSe_3$*/Ag* cross-bar device and (d) another *Ag/*$GeSe_3$*/Ag* cross-bar device. The device is biased for the switching voltage to increase with increasing illumination irradiance. The device undergoes structural damage at higher intensities, resulting in the optically driven voltage shifts to decrease.

Supplementary Section S5

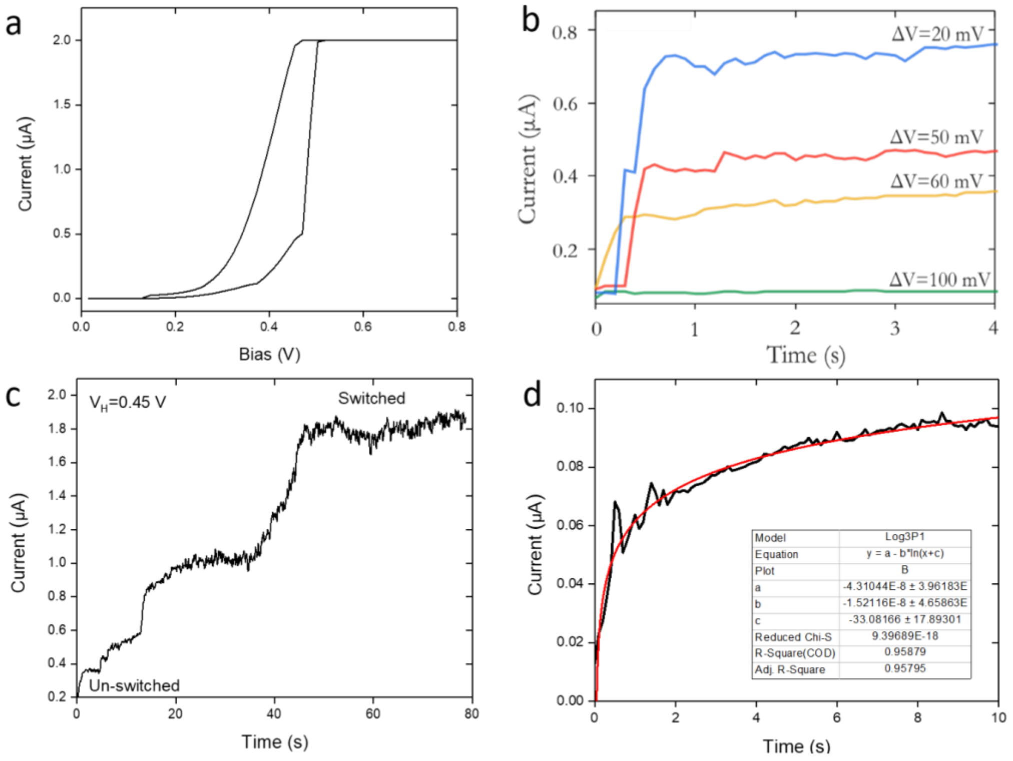

Supplementary Figure S5.1. (a) Current-voltage characteristics of a *Pt/GeSe₃/Ag* cross-bar device. (b) Dynamic switching behavior of the device biased at varying voltages relative to the switching voltage. (c) The device undergoes multi-stage switching events from the high resistance state into the low resistance state when biased at a holding voltage of 0.45 V. (d) The switching event in the most instance is preceded by a decrease in the device resistance that follows the increase in the current as a function of time. The dependency is fitted with a logarithmic function ($I = a - b \times ln(t + c)$) as traditionally observed in filamentry memristors (*Nat. Nanotechnology 2020,15,574*), where a, b and c are fitting constants and I and t are current and time, respectively.

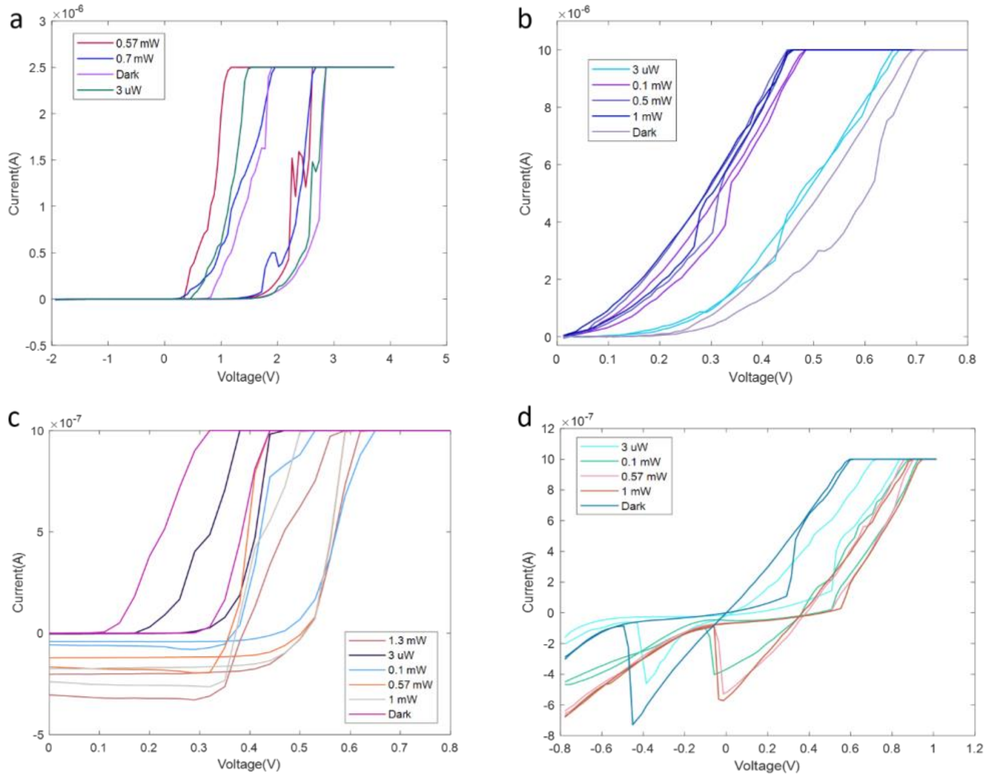

Supplementary Figure S5.2. Current-voltage characteristics of more cross-bar devices under varying illumination conditions. (a) An *ITO/GeSe₃/Ag* cross-bar device. The switching voltage decreases with increasing illumination irradiance. (b) A *Pt/GeSe₃/Ag* cross-bar device with a higher compliance current. The switching voltage decreases with increasing illumination irradiance. (c) Another *Pt/GeSe₃/Ag* crossbar device. (d) An *Ag/GeSe₃/Ag* cross-bar device. The switching voltage increases with increasing illumination irradiance in the devices.

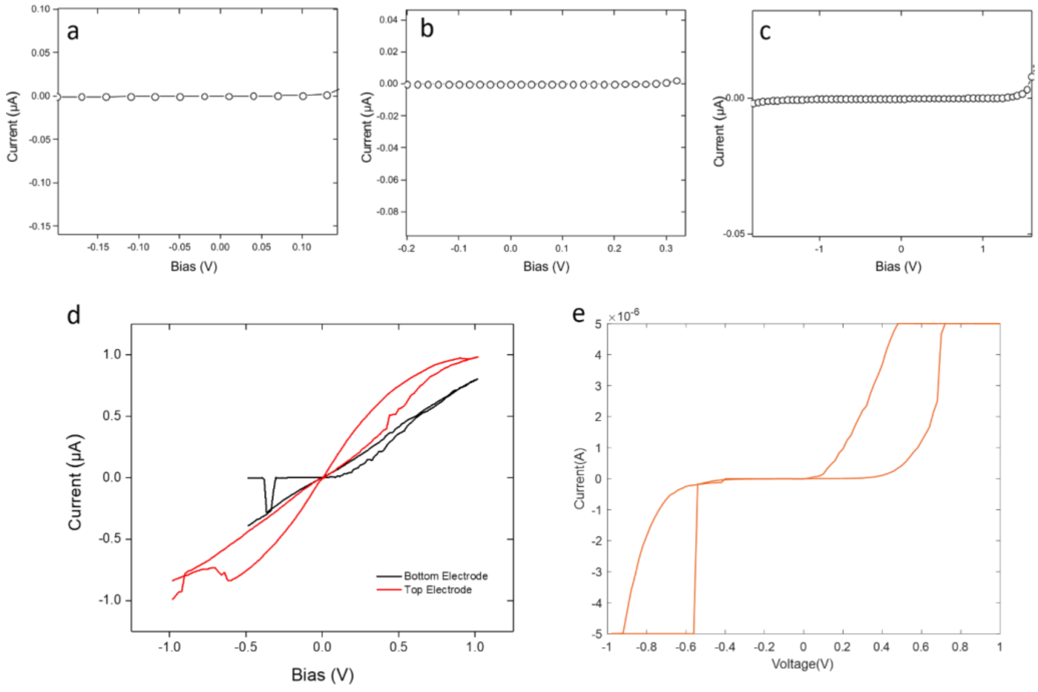

Supplementary Figure S5.3. Current-voltage characteristics of devices in their high resistance state, and at lowfield regime: (a) *Ag/GeSe₃/Ag*, (b) *Pt/GeSe₃/Ag* and (c) *Ag/GeSe₃/ITO*. The IV traces are symmetric suggestive of insignificant diode characteristics. (d) Current-voltage characteristics of an

Ag/GeSe$_3$/Ag device first operated with the bottom-electrode as the ground and subsequently with the top electrode as the ground. (e) Current-voltage characteristics of a *Pt/GeSe$_3$/Ag* device with the top electrode and bottom electrode acting as a ground during the voltage scan.

Supplementary Section S6

In our demonstrations, the total power consumption in implementing three-factor RL equals E_T=N×E_e+×E_o, where N is the number of synapses, E_e is the energy in the electrical signal and E_o is energy in the optical signal. Assuming the mouse takes a new step every other second, a total of t=5 secs are spent in finding the cheese during the course of which in our implementation the eligibility traces are flagged for 3 s. Thus, E_o=s×P_0 mW, while E_e=N×500ns×P_e, where P_0=0.13 mW, and P_0=0.5μA×0.4VmW. E_T=0.39mJ+4×10fJ=≈0.39 mJ. Thus, the energy dominating stimuli is the optical energy, which can be potentially lowered with the engineering of the resonating cavities, smaller exposure times, and the use of more absorptive chalcogenide materials. Compared to emerging memristive hardware approaches (2nd IEEEInternational Conference on Artificial Intelligence Circuits and Systems (AICAS), 218–222 (IEEE, 2020)), there is an order of reduction in energy consumption with the current unoptimized devices. Compared to CMOS, besides a small device footprint, the implementation of the three-factor learning rules with eligibility traces per synapse does not require complex memory structures for and wiring. In contrast, both computation and weight update occur in-place through multi-factorial computation. Compared to the memristive (phase-change memory) approach, the weight update process does not require read-verify cycles, as we employ the physics of optoelectronics to in place combine reward and eligibility traces. Besides, the time scales for computation are tunable in our devices to a wide degree. These, however, are fixed in PCMs thus limiting the realization of vastly different learning applications (*2021 IEEE International Symposium on Circuits and Systems (ISCAS)* 1–5 (2021). doi:10.1109/ISCAS51556.2021.9401446). We however wish to emphasize our motivation is to demonstrate use cases of optomemristors for some higher-order neuronal processes. Within the purview of RL, the implementation of the three-factor learning rules with eligibility trace per synapse in CMOS requires complex memory structures for keeping track of the eligibility trace and the weight. It is the interest of future work to scale up the approach. A discussion on how this could be done is provided in Figure S6.

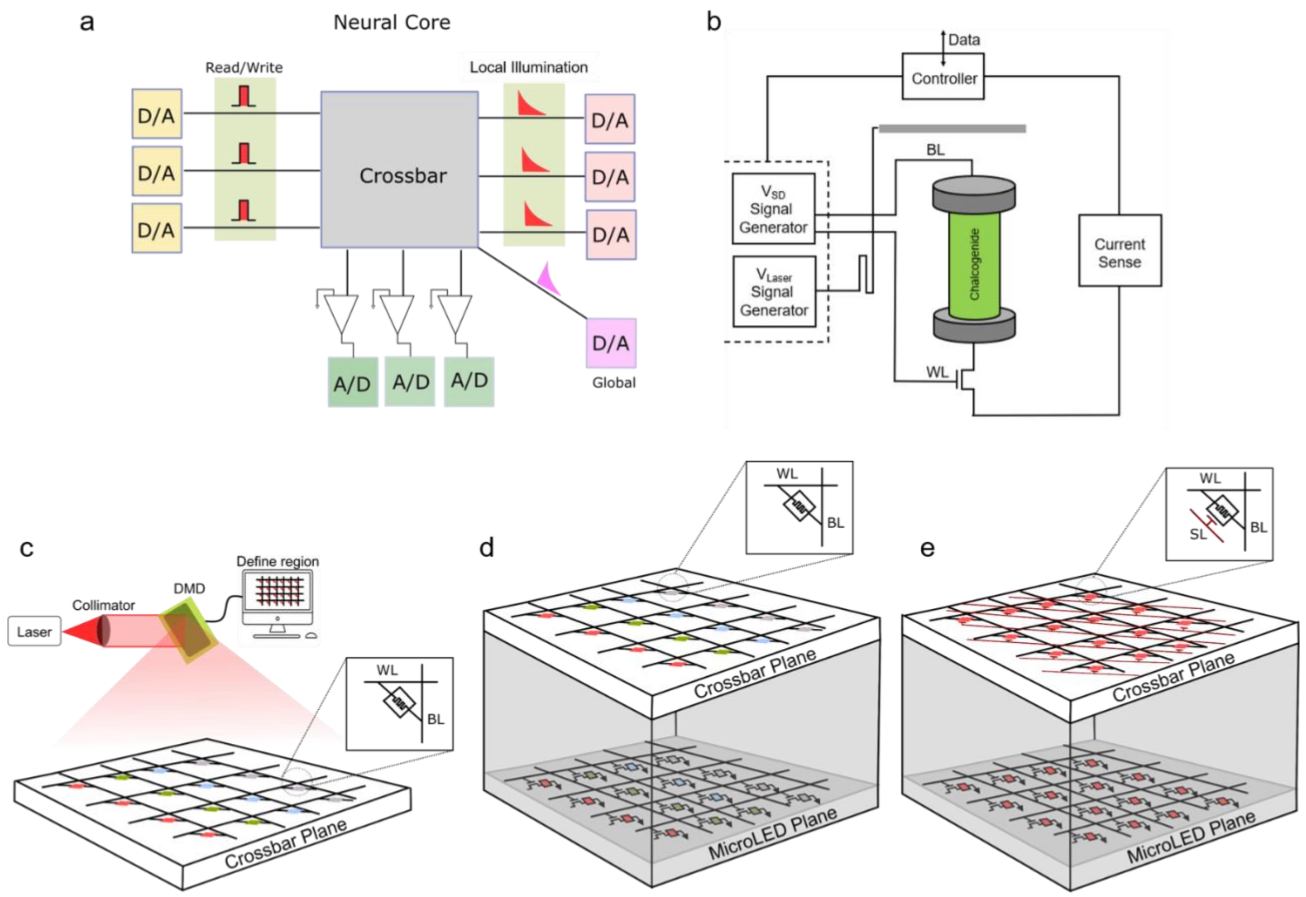

Figure S6. (a) When discussing scaling-up, individual optomemristive devices can be configured into crossbar topologies and be provided with supporting peripheral circuitries. D/A are digital to analog converters for input electrical and optical signals and A/D are analog to digital converters for the current sense. (b) In a crossbar of n by m optomemristive devices (synapses) each device is placed at the crosspoint between word (WL) and bit (BL) lines. The read and write voltage signals (V_n) are applied along the rows (WL) of the crossbar, and the current is measured across the columns (BL). For a given V_n, the current is a function of the non-volatile device conductance ($G_{n,m}$) that can be modulated using both electrical and optical write operation. These states represent the non-volatile states, and for reinforcement learning, for example, these are adjusted only during the training phase. (c) An illustration of the free-space setup for electro-optical modulation of synaptic weights for three-factor learning. Here, the optomemristive devices are electrically stimulated using on-chip circuitries, but optically using free-space lasers. In three-factor learning, for example, since at a given time single or a group of devices on the crossbar must see optical exposure the setup is provided with a digital micromirror device (DMD), that can expand and project a collimated laser beam (Practical holography XIII, vol. 3637,12–20 (International Society for Optics and Photonics, 1999)) of the desired pattern and orientation onto the chip. The DMD is controlled by a standard computer that communicates with the crossbar microcontroller and redirects the beam only to selected optomemristive devices. (d) A fully integrated on-chip architecture utilizing three-dimensional micro-LEDs laid in a crossbar topology. In this approach, the selectivity to expose only selected or a group of devices to light is established by fabricating micro-LEDs that emit distinct wavelengths (Nature Nanotechnology1–6 (2021)), and correspondingly fabricating optomemristive cavities that sense distinct wavelengths using stack thickness engineering. Here, each column (BL) of the micro-LED crossbar plane comprises LEDs that emit distinct wavelengths, i.e. all devices in the same column emit the wavelength of light and aligned on the top plane are optomemristive devices, which on the same column (BL) of their crossbar sense the same wavelength of light. Thus to trigger different columns of the crossbar, corresponding micro-LEDS columns must be turned on. (e) So far we discussed optomemristive devices which used optical

signals for eligibility traces and electrical signals for a reward for three-factor learning. In an alternate approach, the electrical signal can be used as an eligibility trace and vice versa. One approach is to use the semiconductivity of the chalcogenide glasses by using a three-terminal configuration for the optomemristors. Here the temporal modifications to the conductance at the level of individual synapses are achieved using the gate signal (V_{Gate}), which is applied to the synapses using the diagonal selector lines (SL). A gate selector line modifies the Fermi level in the chalcogenide channel and through it modifies the temporal conductance. Note that the diagonal connections allow for parallel modulations, such that multiple devices can be modified simultaneously. In an integrated setup, the reward signal can be applied using single micro-LEDs or an array of micro-LEDs, all emitting light of the same wavelength. Only devices that are electrically stimulated using SL lines will undergo a switching event. This approach however requires that the chalcogenide can be electrostatically tuned, thus requiring the need for low band-gap and low carrier concentration materials.

Supplementary Section S7

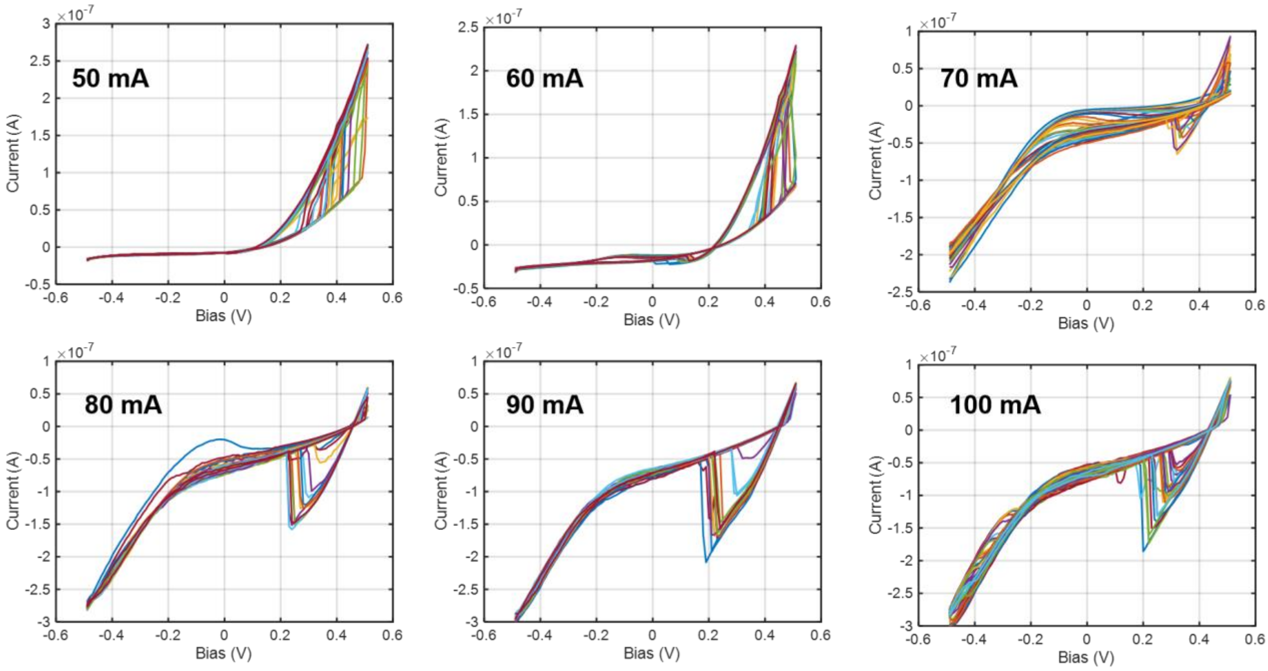

Supplementary Figure S7.1. Additional data. Current-voltage traces under different illumination conditions of a Ag/$GeSe_3$/Ag device. The optical power is defined by the drive current to the laser diode.

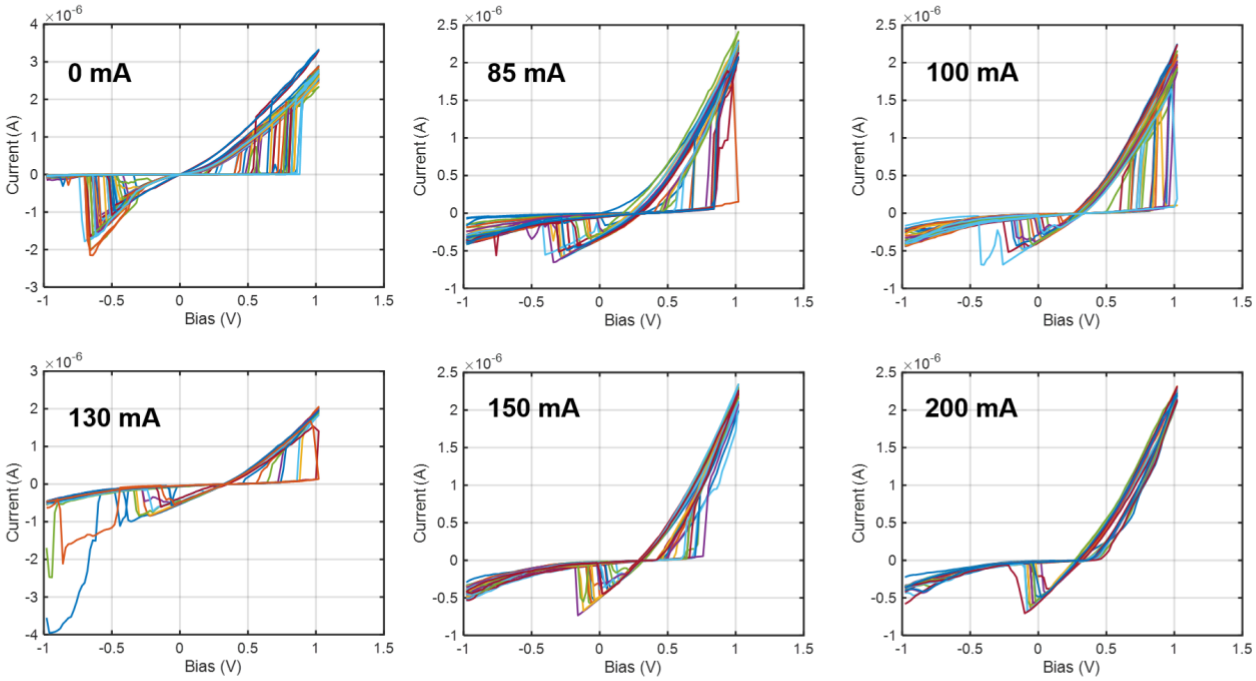

Figure S7.2. Additional data. Current-voltage traces under different illumination conditions of another Ag/$GeSe_3$/Ag device. The optical power is defined by the drive current to the laser diode.

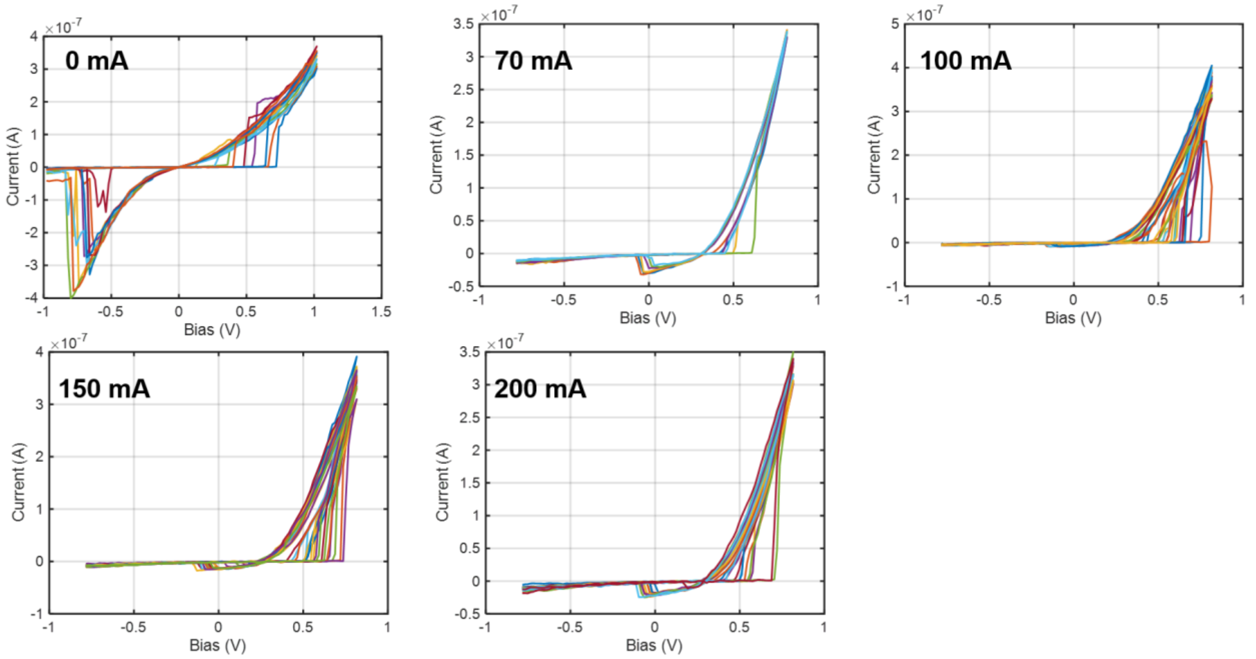

Figure S7.3. Additional data. Current voltage traces under different illumination conditions of another Ag/$GeSe_3$/Ag device. The optical power is defined by the drive current to the laser diode

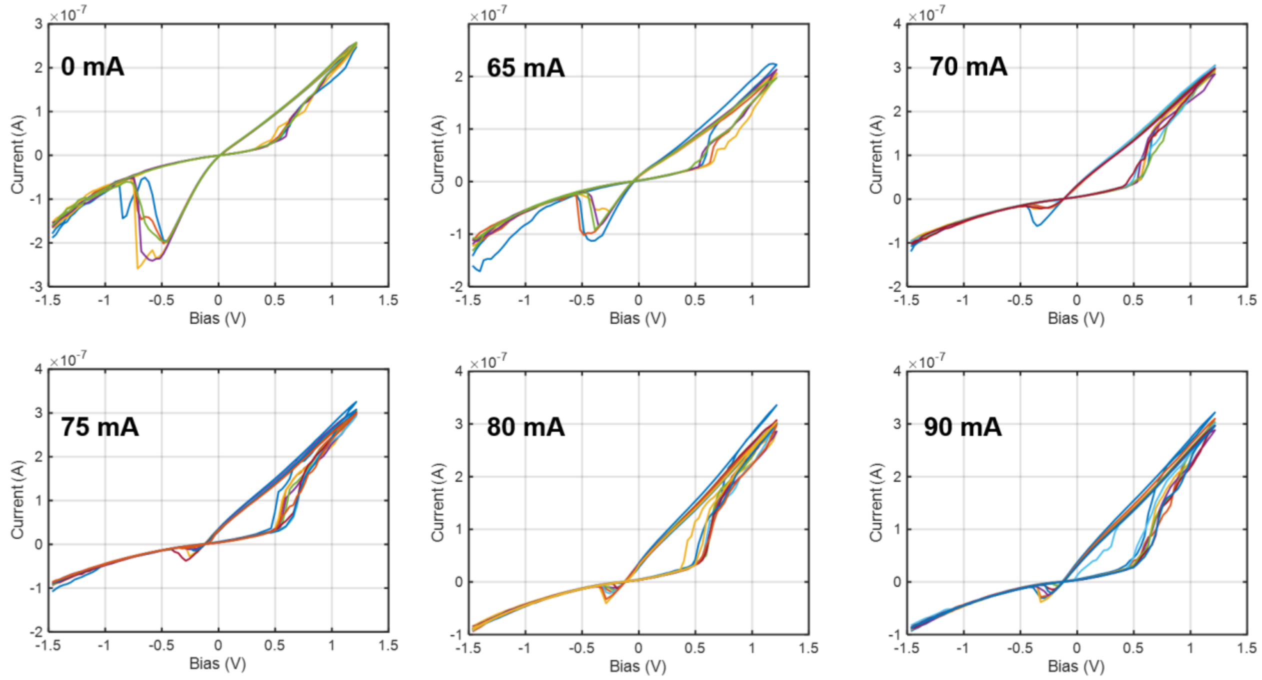

Figure S7.4. Additional data. Current voltage traces (under reverse polarity and under different illumination conditions) of another Ag/$GeSe_3$/Ag device. The optical power is defined by the drive current to the laser diode

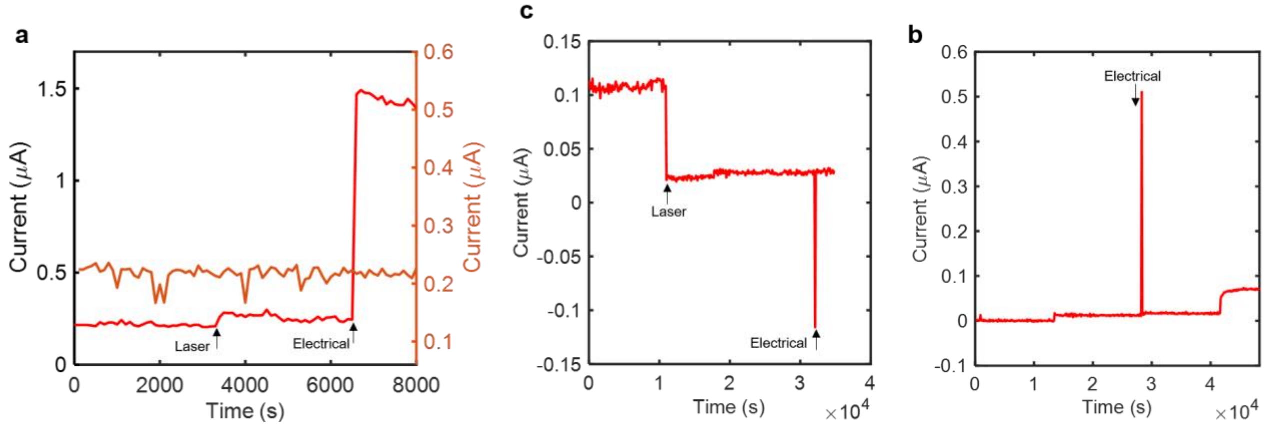

Figure S7.5. (a) Red trace shows mixed-mode switching in a Pt/$GeSe_3$ device. The orange trace is the dark current in the device, where switching events are absent. (b) Same experiment as in (a) but in the reverse polarity. An electrical pulse does not induce aswitching event in when the device is illuminated. (c) Same experiment as in (a) but when the device is biased at a lower holding voltage, andwhen there is no illumination. An electrical pulse fails to induce a switching event
